# Contribution of statistical learning in learning to read across languages

**DOI:** 10.1371/journal.pone.0298670

**Published:** 2024-03-25

**Authors:** Jinglei Ren, Min Wang

**Affiliations:** Department of Human Development and Quantitative Methodology, University of Maryland, College Park, Maryland, United States of America; Education University of Hong Kong, HONG KONG

## Abstract

Statistical Learning (SL) refers to human’s ability to detect regularities from environment Kirkham, N. Z. (2002) & Saffran, J. R. (1996). There has been a growing interest in understanding how sensitivity to statistical regularities influences learning to read. The current study systematically examined whether and how non-linguistic SL, Chinese SL, and English SL contribute to Chinese and English word reading among native Chinese-speaking 4th, 6th and 8th graders who learn English as a second language (L2). Children showed above-chance learning across all SL tasks and across all grades. In addition, developmental improvements were shown across at least two of the three grade ranges on all SL tasks. In terms of the contribution of SL to reading, non-linguistic auditory SL (ASL), English visual SL (VSL), and Chinese ASL accounted for a significant amount of variance in English L2 word reading. Non-linguistic ASL, Chinese VSL, English VSL, and English ASL accounted for a significant amount of variance in Chinese word reading. Our results provide clear and novel evidence for cross-linguistic contribution from Chinese SL to English reading, and from English SL to Chinese reading, highlighting a bi-directional relationship between SL in one language and reading in another language.

## Introduction

Developing sensitivity to orthographic regularities is an important process in reading acquisition (e.g., [1]). Previous research suggests that children’s sensitivity to orthographic regularities relies on statistical learning (SL) ability (e.g., [2]). SL is defined as human beings’ ability to detect regularities from environment (e.g., [3, 4]). There has been a growing interest in understanding statistical properties of mapping between letters and sounds and how sensitivity to these statistical regularities influences learning to read (e.g., [5, 6]). SL ability can affect reading ability both by enabling discovery of probabilistic grapheme-sound correspondences and by boosting linguistic resources such as vocabulary [7]. The present study systematically examined contribution of non-linguistic and linguistic SL in reading first language (L1) Chinese and second language (L2) English across late elementary and middle school grades. Given that Chinese and English have distinct visuo-orthographic structures and statistical regularities, the focus on SL in Chinese L1 and English L2 offers a great opportunity for investigating the contribution of SL to learning to read L2 across different writing systems.

### SL across domains and modalities

Learning is often regarded as a process of following rules that are taught explicitly. For instance, children are taught the order of operations in math. During this process, children are able to realize and describe the rules that they learn. However, in some other cases, learners learn the probabilistic patterns of things implicitly other than all-or-none rules. That is to say, they learn incidentally without a clear awareness of what they have learned. The term SL is broadly used to characterize incidental learning about the regularities among things and events in the environment [6]. SL was initially discovered in infants’ ability to segment speech. Saffran et al. [4] conducted a study in 1996 where they exposed 8-month-old infants to a synthesized speech stream consisting of four three-syllable "words" with 12 unique syllables in random order. The only indication of word boundaries was the transitional probabilities (TPs) between syllable pairs. For instance, a TP of 1.00 was assigned to *tu-pi* as *pi* always followed *tu* within the word *tupiro* (a within-word syllable pair). In contrast, a TP of 0.33 was given to *ro-go* as *golabu* was one of three words that could follow *tupiro* (a between-word syllable pair). After only two minutes of exposure, the infants demonstrated that they had learned the sequential pattern. Saffran et al.’s (1996) groundbreaking study and subsequent studies have demonstrated that people can extract probability information from language. This led to a question of whether statistical learning (SL) is exclusive to language or if it also applies to non-linguistic stimuli. Saffran et al. [8] replicated their previous study to investigate infants’ ability to detect statistical regularities in tone stimuli. Infants were able to identify statistical patterns in sequences of non-linguistic tones, suggesting that statistical structures can be extracted from auditory input regardless of whether it is speech syllables or non-linguistic tones.

It is worth noting that Saffran and colleagues’ studies [4, 8] focused on auditory input, but subsequent research has shown that statistical learning (SL) also occurs across different modalities. For instance, Kirkham and colleagues [3] conducted a study using visual stimuli with infants of different ages (2, 5, and 8 months), where the stimuli were organized in a statistically predictable pattern. Infants were able to detect changes in the pattern and showed greater interest in novel stimuli, indicating the presence of a modality-general SL even in very young infants. Researchers have also extended SL to the visual modality in adult research, such as Fiser and Aslin’s [9] visual triplet paradigm. In this paradigm, participants were presented with sequences of shapes organized into temporal triplets. They were able to distinguish sequences presented during familiarization from both novel sequences of familiar shapes and sequences of shapes seen during familiarization but presented less frequently.

In summary, there is ample evidence indicating that SL operates across various modalities and domains (e.g., [3, 8, 10]). However, regarding investigating the relationship between SL and reading, previous studies have typically viewed SL as a general construct that applies to all domains and modalities. There has been a general belief that all SL computations are controlled by a central mechanism and are equally important in all aspects of linguistic performance (e.g., [11]). Some recent studies have observed different magnitudes of correlation between SL and linguistic-related measures as a result of different types of SL tested varying its domain and modality, some researchers have challenged the domain-general view of SL and called for a more nuanced understanding of the specific computations underlying SL (e.g., [11–14]).

### Contribution of SL to reading in an alphabetic writing system

In the handful of studies that have examined how SL relates to reading in alphabetic writing systems, some studies examined domain-specific SL effects in alphabetic languages (e.g., [15–19]), and some other studies focused on domain-general SL effects. Among these studies, some revealed positive relationships between visual SL (VSL) and word reading, and between auditory SL (ASL) and word reading (e.g., [20–23]). Arciuli and Simpson [20] utilized the classic triplet paradigm to test non-linguistic VSL, and the reading subtest of the Wide Range Achievement Test (WRAT-4; [24]) to assess children’s ability to read aloud individually presented English orthographic strings. A positive relationship was revealed between non-linguistic VSL and reading ability in children and adults. Furthermore, Torkildsen et al. [23] used the same paradigm and showed that Norwegian school-aged children’s performance on a non-linguistic VSL task predicted a unique amount of variance in real word reading efficiency in Norwegian.

Not only is VSL related to reading, but also ASL contributes to reading development. Some researchers proposed that ASL plays a stronger role in certain aspects of reading compared to VSL probably because ASL is likely related to phonological awareness, essential for decoding. Spencer et al. [25] assessed linguistic ASL using the classic triplet paradigm and non-linguistic VSL using a Simon task among native English-speaking children, as well as a number of reading-related measures such as phonological awareness and morphological comprehension tests. In the Simon task, participants were presented with sequences of four colored shapes and then were asked to reproduce the sequence of colors in the current order. Results showed that both linguistic ASL and non-linguistic VSL were related to reading tasks, but linguistic ASL was more closely associated with these tasks than VSL. Note that their linguistic ASL and non-linguistic VSL varied in both domain and modality, hence the two effects could not be teased apart. Furthermore, Qi et al. [21] measured children’s and adults’ performance on non-linguistic ASL (tone triplet paradigm) and non-linguistic VSL (classic triplet paradigm) and their performance on real word, non-word, and sentence reading. Results showed that non-linguistic ASL, but not non-linguistic VSL was significantly associated with sentence reading fluency in the combined sample of children and adults. One possible explanation for this finding is that phonological awareness is a potential mediator between ASL and reading acquisition, namely, ASL contributes significantly to phonological awareness and phonological awareness plays an important role in reading acquisition.

However, some other studies did not find significant association between SL and reading. For example, Schmalz et al. [26] tested German speaking adults on two non-linguistic VSL tasks (serial reaction time SRT and artificial grammar learning AGL), word and nonword reading fluency tasks. No significant association was shown between SL tasks and reading ability.

Given that previous studies have observed different magnitudes of association between different types of SL and reading-related measures, some researchers suggest that the different types of SL computations may not be equally important in reading performance. These researchers have called for a refinement of SL theory, which should be thought of as a componential, rather than a domain-general construct ([11, 12, 14, 27]). Importantly, while these researchers differ in their exact theoretical approaches, they all share one important principle—SL involves a set of non-overlapping underlying computations. Thus, they all call for a better understanding of the nature of domain-general versus domain-specific computations in SL, accompanied by explicit mapping of its sub-components. If SL is a componential ability rather than a unified construct, a single task cannot cover the full scope of SL computations, the current study thus took a comprehensive approach to systematically examine how SL across domains and modalities contributed to reading skills using comparable SL tasks (i.e., linguistic visual, linguistic auditory, non-linguistic visual and non-linguistic auditory).

### SL in a non-alphabetic writing system

Limited research has investigated how SL plays out in a nonalphabetic writing system. Most have focused on SL in Chinese, a representative non-alphabetic writing system. Given the Chinese writing system’s unique orthography and its statistical regularities, it provides a fascinating opportunity for investigating visual SL and its underlying mechanisms. The basic orthographic unit in Chinese is a character that represents a syllable and a morpheme, and this orthographic feature is dramatically different from English and other alphabetic orthographies. Each Chinese character is composed of basic strokes, which are combined to form a square-like block. Left-right (e.g., 静[jìng],*quiet*) and top-bottom (e.g., 花[huā], *flower*) are the two most common structures in characters’ visuo-orthographic configuration (e.g., [28]). In terms of phonology, Chinese is a tonal language with four main tones and each tone has a distinctive pitch contour. A syllable segment with a different tone represents a different meaning, for example, 妈 ([mā], tone 1) refers to *mother*, 麻 ([má], tone 2) to *hemp*, 马 ([mă], tone 3) to *horse*, and 骂 ([mà], tone 4) to *scold*.

Sub-lexical regularities lay the foundation for recognizing individual words rapidly and accurately, which, in turn, facilitates fluent reading of phrases and sentences (e.g., [29, 30]). As readers become more adept at recognizing words by sight, they can focus on understanding the overall meaning of the text and comprehend phrases and sentences more efficiently. Over 80% of Chinese characters are created by combining a semantic radical, which provides a hint to the character’s meaning, with a phonetic radical, which provides a hint to the character’s pronunciation [28]. For instance, the character 枝(branch) /tʂɨ1/ is made up of the semantic radical 木(wood), indicating a wood-related concept, and the phonetic radical 支/tʂɨ1/, providing a cue to the pronunciation of the whole character. There are certain positional regularities among Chinese radicals, such as phonetic radicals usually appearing on the right or bottom position and semantic radicals often appearing on the left or top position, in left-right or top-bottom structured characters, respectively (e.g., [28]). Additionally, most Chinese words are made up of two or three characters, such as the word for ’train,’ which is 火车 (huǒ chē), literally meaning "fire car." Similarly, a helicopter—直升机 (zhí shēng jī)—is a "straight-rising machine." The sequential patterns between characters provide helpful information in determining the meanings of Chinese words.

Yin and McBride [2] conducted one of the earliest studies on Chinese children’s ability to learn positional consistencies. They found that 5-year-old children in China performed better in a task where they had to identify legally positioned radicals within Chinese characters compared to when the same radicals were positioned illegally. This suggests that even before receiving formal instruction, Chinese children are able to identify the statistical patterns in Chinese characters. Similarly, He and Tong [31] showed that children in grades 3–5 could differentiate novel characters that had radicals in legal positions from those that did not after a short exposure. These studies demonstrate that children are sensitive to orthographic regularities when learning to read Chinese characters. However, these studies focused on within-character statistical patterns. The current study, in contrast, focused on sequential patterns at the word level using spoken syllable and written character triplets to explore children’s sensitivity to between-syllable and between-character sequential regularities. The goal was to determine if this sensitivity contributed to reading Chinese.

### SL in two languages

Previous research has focused on SL in L1. Very limited research examined SL in L2 (except for [32]). Frost et al. [32] focused on native English speakers learning Hebrew L2. The classic triplet visual SL paradigm was adopted, monitoring participants’ implicit learning of the transitional probabilities of visual shapes. Non-linguistic VSL ability was shown to be correlated with Hebrew reading proficiency [32]. That is, the native speakers of English who more accurately picked up the implicit statistical structure embedded in the continuous stream of nonsense visual shapes better assimilated the Semitic structure of Hebrew words.

However, the stimuli used in their study were non-linguistic, thus it is unclear whether linguistic SL in L1 would contribute to reading in L2. Different from nonlinguistic SL which involves more abstract visual stimuli such as images or pictures, linguistic SL involves the processing of statistical regularities embedded in linguistic materials. Moreover, the linguistic materials can vary depending on different languages that we have Chinese and English SL materials in our study. In the current study via a classic triplet paradigm, we examined linguistic SL of syllable sequential patterns from a stream of linguistic auditory and visual materials. We were interested in investigating whether linguistic and non-linguistic SL both contribute to reading in L1. We were also interested in whether the cross-linguistic transfer would occur between SL in L1 and reading in L2 or vice versa, between SL in L2 and reading in L1. Chinese L1 and English L2 provide a fascinating opportunity to answer this question due to their distinct visual-orthographic structures. When L1 and L2 present contrasting linguistic and orthographic environments in terms of statistical properties, it is interesting to see whether and how cross-linguistic influence occurs. Previous research has provided ample evidence supporting for an interactive transfer from reading-related skills such as phonological and morphological awareness in L1 to reading outcomes in L2 across similar languages and orthographies (e.g., French-English, [33]) as well as those that are distinct (e.g., Chinese-English, [34–36]). Our current study extends the previous research on cross-language transfer to investigate whether and how SL, an implicit cognitive skill, can be transferred across two different writing systems.

### The present study

We examined whether non-linguistic VSL and ASL, linguistic Chinese VSL and ASL, and linguistic English VSL and ASL contribute to Chinese and English reading abilities among native Chinese-speaking grades 4 (around 9-year-old), 6 (around 11-year-old), and 8 (around 13-year-old) children acquiring English L2. We employed six SL tasks, two of them examining non-linguistic VSL and ASL, two examining Chinese VSL and ASL, and the remaining two examining English VSL and ASL. Note that the data for the SL measures has been published in [37], which focused on the developmental trajectory of SL but did not address the association between SL and reading.

We addressed the following questions: 1) Does non-linguistic SL contribute to Chinese and English word reading? 2) Does linguistic SL contribute to word reading within English or Chinese? 3) Is there any cross-linguistic contribution, that is, whether Chinese SL contributes to English word reading, or English SL contributes to Chinese word reading? 4) Do visual and auditory SL differentially contribute to Chinese and English word reading? Based on previous evidence (e.g., [20, 21]) on the important role of non-linguistic VSL and ASL in reading English, we hypothesized that non-linguistic VSL and ASL would contribute to reading in both Chinese and English in the current study. For linguistic SL, we hypothesized that Chinese VSL would make a greater contribution to Chinese reading compared to Chinese ASL, since learning to read Chinese may rely more on visual-orthographic compared to phonological information (e.g., [38]). Furthermore, English VSL and ASL would both contribute to English reading, since reading English requires sensitivity to patterns of mapping between sounds and letters. For cross-linguistic contribution, Chinese VSL may not contribute to English reading and English VSL may not contribute to Chinese reading given the sharp contrast in visual-orthographic statistical regularities between Chinese and English writing systems. However, Chinese ASL and English ASL may contribute to English and Chinese reading, respectively, given the shared syllable segmental structure.

## Method

### Participants

Ethics approval (#1683348–1) was obtained from the Internal Review Board (IRB) at the University of Maryland College Park. Written Informed consent was obtained from parents. We used participant IDs and did not ask for names so we did not have access to information that could identify individual participants during or after data collection. Forty fourth (mean age = 9.2, standard deviation = 0.41, male = 17), 40 sixth (mean age = 11.1, standard deviation = 0.62, male = 15), and 40 eighth graders (mean age = 13.5, standard deviation = 0.38, male = 18) were recruited from a primary school in Yantai, China from March 2021 to July 2021. All participants were native Chinese speakers and had at least one year of formal English instruction by age 9. The starting age of 9 ensured that our participants had some English language proficiency to allow them to perform the English tasks. Parents were asked to fill out a home language experience questionnaire. Eighty-seven percent of children started to learn English in grade 3. Seventy-two percent of children only spoke English in English classes while the others received private lessons after class. We also asked three English teachers to fill out a questionnaire regarding their teaching experiences and approaches to English instruction. Results showed that all the three teachers achieved high scores in standard English proficiency tests: one of them achieved CET (College English Test)-Level 6, while the other two achieved TEM (Test for English Major)-Level 6. Each English class is 45 minutes long, and children have 5 lessons each week. In addition, all the teachers apply whole-language based approach in teaching English. Two teachers ranked vocabulary and grammar as the most important in teaching English, while the other one ranked reading as the most important. Only one of them sometimes emphasized rules/patterns when teaching English in class, and the other two almost never emphasized the importance of rules/patterns.

### Measures

#### SL tasks

**Fig 1:**
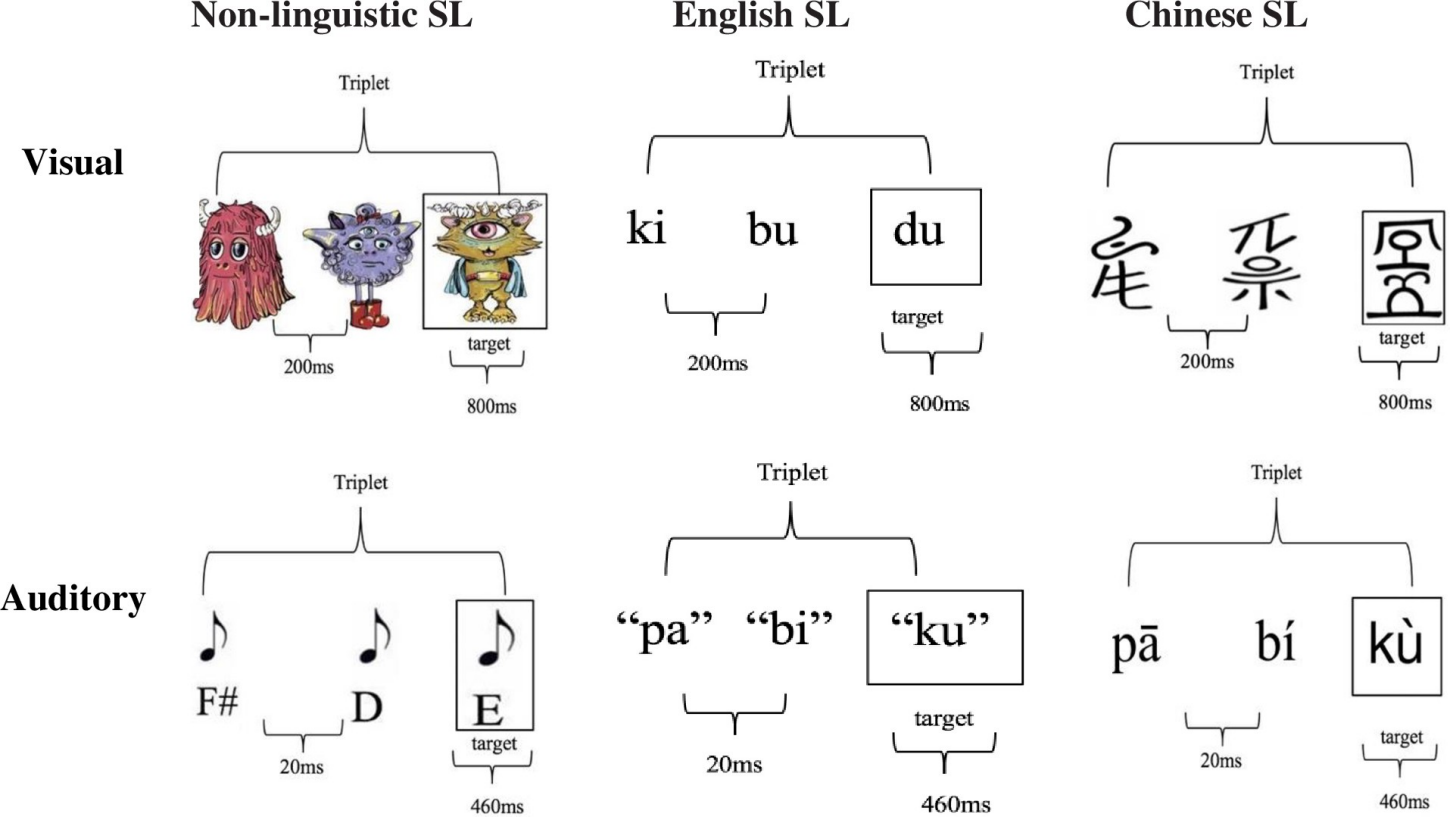
Familiarization phase demonstration of all six SL tasks. *Note*. Example triplets across each task are depicted in this figure. Each visual stimulus appeared for 800ms with a 200ms interval, and each auditory stimulus was heard for 460ms with a 20ms interval.

Following the classic triplet paradigm [39], all of the SL tasks included a familiarization and a testing phase. In the familiarization phase, children either saw or heard a sequence of images, syllables, characters, or tones and were asked to track a specific target (see Fig 1, [37]). Within each triplet, the stimuli always followed each other. Between triplets, the sequence of stimuli was randomized.

The researcher provided verbal instructions throughout the two phases and monitored to make sure the children stayed on-task.

#### VSL tasks

*Non-linguistic VSL*. This task was adopted from [39] in which 12 unique alien images were divided into 4 groups to form 4 base triplets (ABC, DEF, GHI, and JKL). In the test phase, four new triplets (AEI, DHL, GKC, and JBF) were created using the same alien images. The relative position of each image in a new triplet was the same as that in the base triplet.

Children saw and heard at the beginning “Hi there, today you are going to see some aliens line up to enter a cool spaceship. We need you to help us keep track of a very special alien as the aliens line up to enter their spaceship. We will show you the alien now." In the familiarization phase, we repeated each of the four base triplets 24 times for a total of 96 triplets. Each alien stimulus was presented one at a time on a computer screen for 800 ms. The interstimulus interval was 200 ms. During the familiarization phase, children were instructed to press the spacebar as fast as they could whenever they saw the specific target alien appear on the screen. The target alien image was always the third alien in one of the four base triplets (see Fig 1 upper part for the example of image presentation order). The two alternative forced choice test phase was introduced after the familiarization phase. On test trials, participants were asked to identify which of the two triplets (one was a base triplet and the other was a new one) seemed more like what they saw during the familiarization phase.

*English VSL*. Adapted from [40], 12 visual syllables (ki, bu, du, mo, di, pa, ta, ka, po, lo, ma, ri) formed four triplets (kibudu, modipa, takapo, lomari). The foil triplets were kidipo, moburi, tamapa, lokadu. The design and procedure of the English VSL task was identical to those of the other two VSL tasks. Each English syllable was presented one at a time on a computer screen for 800 ms. The interstimulus interval was 200 ms. Children heard at the beginning “Hi there, we are going to learn a new language today! We need you to help us keep track of a special character as you see different characters appearing on the screen. We will show you the characters now.” See Fig 1 upper part for the example of presentation order of English VSL and Chinese VSL described below.

**Fig 2:**
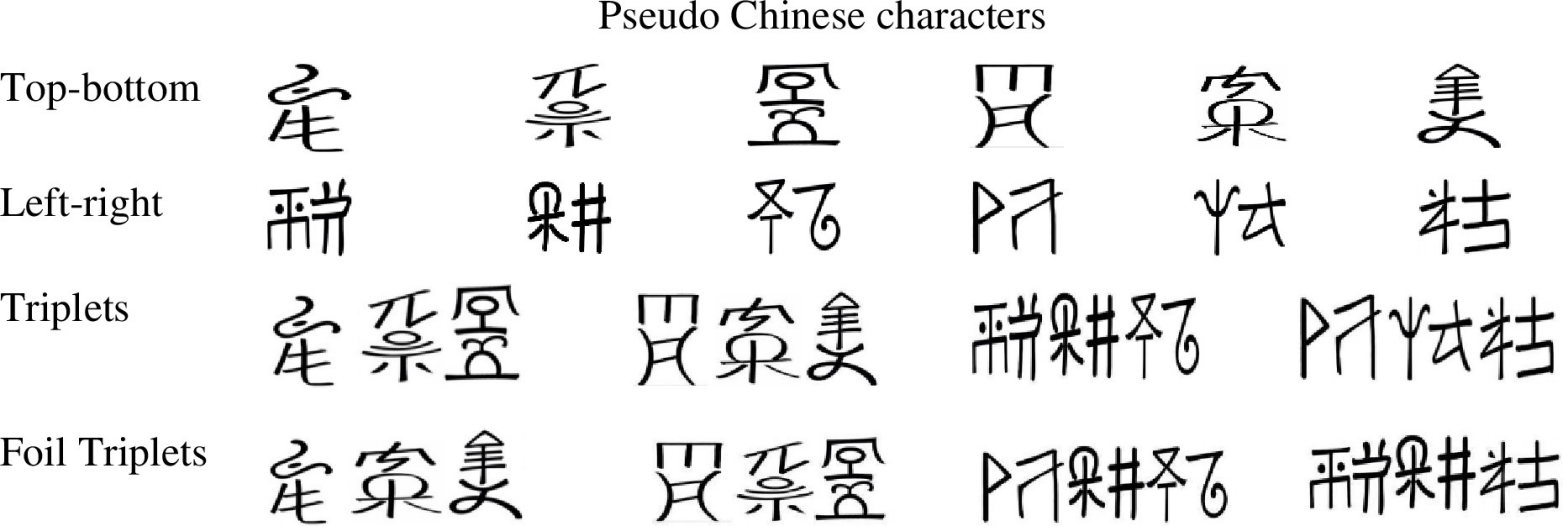
Examples of pseudo-characters in Chinese VSL task.

*Chinese VSL*. We adapted the paradigm used in [31, 41], where 12 pseudo-characters were created by combining two Geba characters (e.g., 
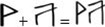
). The Geba characters have their roots in the Chinese loan characters and have a similar appearance to Chinese characters as noted by [42]. They are a great choice for creating fake characters to evaluate how well children can perceive and process the visual and spatial arrangement of characters. Six left-right (e.g., 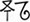) and 6 top–bottom pseudo-characters (e.g., 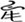) were used, reflecting the two common character structures in Chinese. Each Geba character was presented one at a time on a computer screen for 800 ms. The interstimulus interval was 200 ms. They were divided into four base triplets (see Fig 2, [37]). Chinese VSL’s procedure was the same as the English VSL’s.

The internal consistency of Cronbach’s alphas was .74, .90, and .81 for non-linguistic VSL, English VSL, and Chinese VSL, respectively.

#### ASL tasks

*Non-linguistic ASL*. Modeled after [8], 12 pure tones within the same octave (a full chromatic scale starting from middle C) formed four base triplets (F#DE, ABC, C#A#F, and GD#G#). The new triplets were F#BF, AA#G#, C#D#E, GDC.

Children saw and heard at the beginning "Hi! We’re going to listen to some alien folk music today. We need you to help us keep track of a special sound as you hear different sounds.” During the familiarization phase, we repeated each of the four triplets 48 times for a total of 192 triplets (twice as much as the visual conditions, following the standard paradigm). The duration of each tone is 460 ms with a 20 ms interstimulus interval. Children were instructed to press the spacebar as quickly as possible whenever they heard the target sound in the familiarization phase. The target sound was always the third one of the four base triplets (see Fig 1 lower part for the example of sound presentation order for non-linguistic ASL, English ASL and Chinese ASL tasks described below). In the 2AFC test phase, participants were asked to identify which of the two triplets sounded more like what they heard during the familiarization phase.

*English ASL*. Based on [43], 12 English syllables formed four tri-syllabic words: pabiku, golatu, daropi, and tibudo. The new triplets were gobutu, parodo, tilapi, dabiku. The duration of each English syllable is 460 ms with a 20 ms interstimulus interval. English ASL’s procedure was the same as the non-linguistic ASL.

*Chinese ASL*. We created Chinese auditory syllables comparable to English with four tones evenly distributed. They were pā, bí, kù, gōu, lǎ, tù, dě, ròu, pī, tī, bú, dòu. The four tri-syllabic words were pābíkù, gōulǎtù, děròupī, tībúdòu. The new triplets were gōubútù, pāróudòu, tīlǎpì, děbíkù. The duration of each Chinese syllable is 460 ms with a 20 ms interstimulus interval. The Chinese ASL words were rated by two native Chinese speakers to ensure that the nonwords were non-word like (M = 3.5), rating from 1 to 5, where 1 indicates most word like. Chinese ASL’s procedure was the same as non-linguistic ASL.

The internal consistency of Cronbach’s alphas were .76, .89, and .83 for non-linguistic ASL, English ASL, and Chinese ASL, respectively.

### Reading outcomes

#### English word reading efficiency

This was assessed by the sight word efficiency subtest of Test of Word Reading Efficiency-Second Edition (TOWRE-2nd; [44]). There are 108 words in total, ranging from one syllable to four syllables. Children were asked to read aloud as many words as they could within 45 seconds and skip any words that they could not read. One experimenter marked each item as right or wrong when children read, and children’s pronunciation was recorded. None of the children read all the items. We double checked the recording after the experiment and coded how many English words children read correctly within 45 seconds. The internal consistency Cronbach’s alpha was .91.

#### Chinese word reading efficiency

This was assessed using the two-character Chinese word reading test [45]. There are 180 Chinese words in total. Similar to the English test, Children were asked to read aloud as many words as they could within 45 seconds and skip any words that they could not read. None of the children read all the items. Only if children read both characters in a single word correctly were they coded as correct. We coded how many Chinese words children read correctly within 45 seconds during the experiment and double checked the recording after the experiment. The internal consistency Cronbach’s alpha was .93.

### Control measures

#### English language proficiency

Given that English is our participants’ L2, we measured their vocabulary in English as an index of language proficiency. We used a shortened version of PPVT (the Peabody Picture Vocabulary Test, Fourth Edition; [46]) to assess children’s English language proficiency. We selected 30 items from the original test (items 1–6, 13–18, 37, 39–43, 49–53, 61–64, 73, and 75–76) to ensure the same progression of item difficulty comparable to the original test. The test was administered to participants individually. The experimenter played recording of each item once, and participants were asked to circle the picture in the response booklet that best described the word they heard. Prior to the formal test items, there were four practice items. The internal consistency Cronbach’s alpha was .94.

#### Non-verbal ability

Raven’s Progressive Matrices test (RPM, [47]) was conducted to assess non-verbal ability. The internal consistency Cronbach’s alpha was .87.

#### Procedure

Children were tested individually on a laptop computer. All the SL tasks were written in Jspsych-5.0.3 [48]. There were two sessions, separated by a seven-day interval. In the first session, children completed the TOWRE-2 Sight Word Efficiency subtest [44] and a shortened version of PPVT. After a 5-minute break, children were instructed to take the non-linguistic VSL, Chinese VSL, and Chinese ASL tasks. Task orders were counterbalanced in that half children received reading tasks first, and the other half received SL tasks first. Session one took around 40 minutes. In the second session, children completed the two-character Chinese word reading test and the non-verbal IQ test. Also, children completed non-linguistic ASL, English ASL and VSL tests. Session two took around 45 minutes. Task orders were counterbalanced in session two, similar to session one. Session order was also counterbalanced. Half children received session one first and the other half received session two first. The English word reading test was always alongside Chinese SL tests, and the Chinese word reading test was always alongside English SL tests to minimize the effect of language practice. Instructions for each session were visually displayed on the screen and verbally explained by the experimenter. For Chinese and non-linguistic SL tasks, non-verbal IQ, and Chinese reading measures, instructions were in Chinese. For English SL tasks, English vocabulary, and English reading measures, instructions were in both English and Chinese, to ensure participants’ comprehension of the English task demands.

## Results

The data and original codes are available in Open Science Framework [https://osf.io/3e45m/?view_only=de2df9d61c504129b5d56d9fa12ddc62]. Table 1 presents the descriptive statistics for SL tasks and reading measures.

**Table 1:** Summary of descriptive statistics of all the measurements.

	4^th^ graders 6^th^ graders 8^th^ graders					
	*M*	*SD*	*M*	*SD*	*M*	*SD*
TOWRE-R (words/45s)	18.45	7.25	37.25	12.62	51.08	18.38
CWord (words/ 45s)	70.40	14.04	81.55	18.41	90.20	16.99
Evocabulary (out of 30)	12.55	2.19	15.57	2.83	16.55	3.55
Non-verbal IQ (out of 24)	16.15	3.25	17.98	4.59	20.80	3.56
Non-linguistic VSL	0.62***	0.12	0.67***	0.16	0.81***	0.15
Non-linguistic ASL	0.56***	0.07	0.60***	0.12	0.69***	0.14
Chinese VSL	0.59***	0.12	0.64***	0.13	0.71***	0.15
Chinese ASL	0.62***	0.11	0.64***	0.15	0.73***	0.17
English VSL	0.61***	0.12	0.67***	0.13	0.71***	0.17
English ASL	0.54**	0.07	0.59***	0.11	0.67***	0.15

*Note*. Evocabulary = English receptive vocabulary; TOWRE-R = TOWRE real word reading; CWord = Chinese word reading.

* indicates above change level

**p < .01

***p < .001.

**Fig 3:**
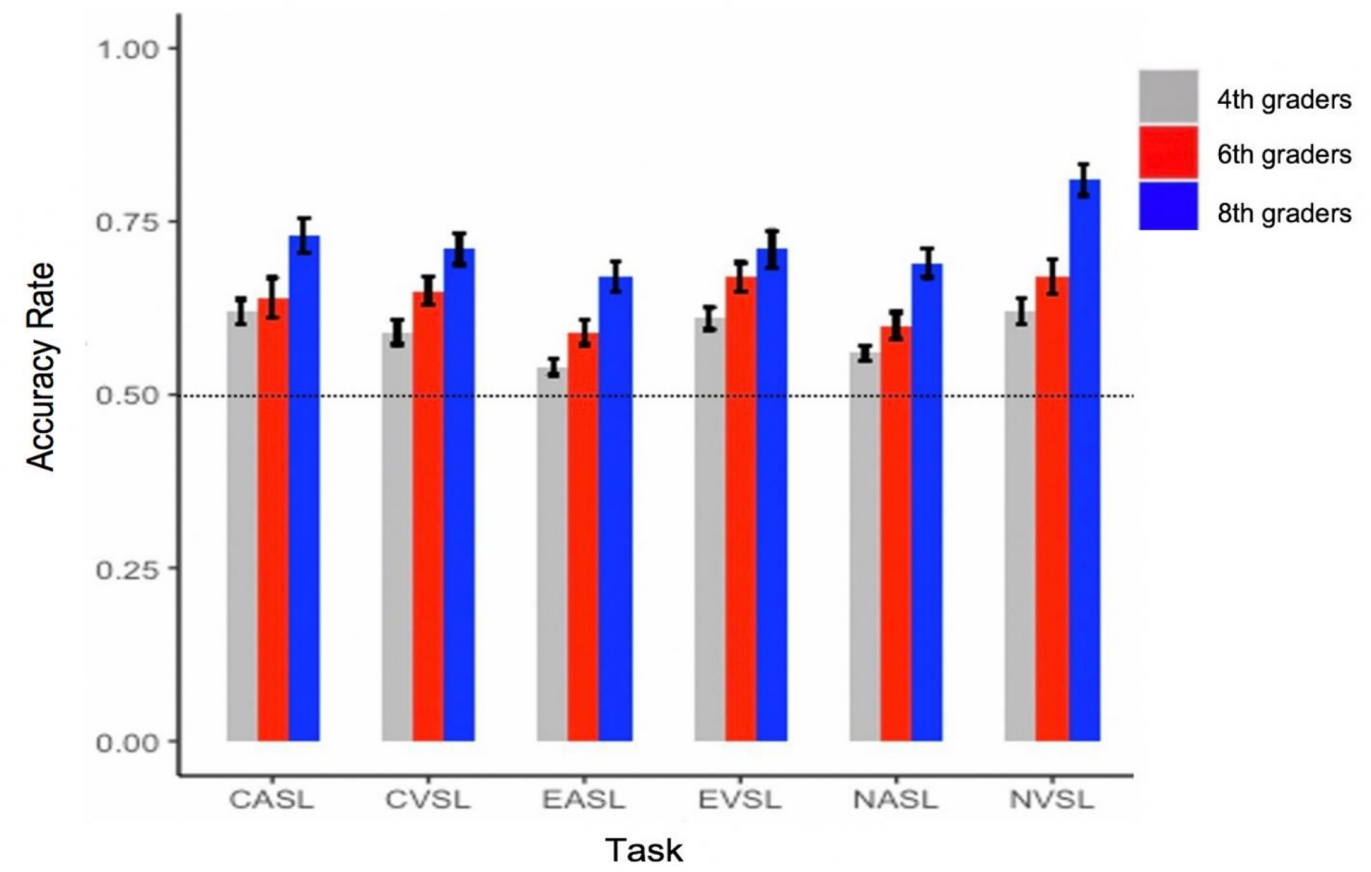
Mean SL accuracy rates across all the tasks by grade. *Note*. Accuracy rates in the 2AFC task. The dotted line represents the chance level. CASL = Chinese ASL; CVSL = Chinese VSL; EASL = English ASL; EVSL = English VSL; NASL = Non-linguistic ASL; NVSL = Non-linguistic VSL.

Children across all grades performed significantly better than chance level (50%) across all SL tasks including non-linguistic VSL and ASL, Chinese VSL and ASL, English VSL, and ASL tasks (all *ps* < .05, see Fig 3).

All participants’ SL accuracy was within 2.5 standard deviations. The bivariate Pearson correlations among all the tasks are shown in Table 2.

**Table 2:** Pearson correlation matrix of statistical learning performance and reading ability in English and Chinese across grades.

	1	2	3	4	5	6	7	8	9	10
1.Grade										
2.Evocabulary	.49***									
3.NV-IQ	.45***	.40***								
4.TOWRE-R	.70***	.71***	.61***							
5.Cword	.44***	.49***	.42***	.65***						
6.NVSL	.46***	.49***	.47***	.63***	.36***					
7.NASL	.43***	.51***	.38***	.64***	.47***	.57***				
8.EVSL	.29**	.42***	.28**	.54***	.42***	.41***	.49***			
9.EASL	.43***	.50***	.29**	.49***	.25**	.42***	.51***	.51***		
10.CVSL	.36***	.42***	.33**	.47***	.44***	.42***	.42***	.39***	.41***	
11.CASL	.30**	.41***	.36***	.51***	.33**	.55***	.42***	.29**	.43***	.52***

*Note*. Evocabulary = English receptive vocabulary; NV-IQ = Non-verbal IQ; TOWRE-R = TOWRE real word reading; CWord = Chinese word reading.

NVSL = Non-linguistic VSL; NASL = Non-linguistic ASL; EVSL = English VSL; EASL = English ASL; CVSL = Chinese VSL; CASL = Chinese ASL.

**p < .01

***p < .001.

All the SL tasks were significantly correlated with Chinese and English word reading. Grade, English receptive vocabulary, and non-verbal IQ were significantly correlated with all the SL tasks and reading measures.

### Predicting English word reading

To examine the unique contribution of SL in English reading, we ran a hierarchical linear regression model through the lm function [49] in R software [50]. Grade was center-coded, and English word reading was entered as the outcome variable. Grade, nonverbal IQ, and English receptive vocabulary were entered as control factors in the first step. In the second step, we entered non-linguistic VSL and ASL as a block to examine whether non-linguistic SL contributes to English reading. In the third step, we entered English VSL and ASL as a block to examine whether English SL contributes to English reading independent of non-linguistic SL. In the final step, to examine whether Chinese SL makes a cross-linguistic contribution to English reading beyond non-linguistic SL and English SL, we entered Chinese VSL and ASL as a block (see Table 3).

**Table 3:** Summary of hierarchical regression analysis for variables predicting performance on English word reading efficiency.

Variables	*ß*	*SE*	*t*	*R*	*R* ^*2*^	Δ*R*^*2*^
**Step 1**				0.85	0.73	0.73
grade	0.35	0.67	6.51***			
English vocabulary	0.30	0.32	7.43***			
Non-verbal IQ	0.19	0.24	4.82***			
**Step 2**				0.88	0.78	0.05
NVSL	0.05	6.89	1.89+			
NASL	0.16	8.67	3.39***			
**Step 3**				0.90	0.81	0.03
EVSL	0.18	6.91	3.22**			
EASL	-0.10	8.56	-1.35			
**Step 4**				0.92	0.84	0.03
CVSL	-0.02	7.22	-0.33			
CASL	0.12	7.11	2.14*			

*Note*. NVSL = Non-linguistic VSL; NASL = Non-linguistic ASL; EVSL = English VSL; EASL = English ASL; CVSL = Chinese VSL; CASL = Chinese ASL.

^+^*p* < .1

*p < .05

**p < .01

***p < .001.

Non-linguistic ASL made a unique contribution after controlling grade, nonverbal ability, and English receptive vocabulary (*β* = .16, *SE* = 8.67, *t* = 3.39, *p* = .001). English VSL accounted for a unique amount of variance beyond non-linguistic SL (*β* = .18, *SE* = 6.91, *t* = 3.22, *p* = .002). Chinese ASL contributed a significant unique amount of variance beyond English SL (*β* = .12, *SE* = 7.11, *t* = 2.14, *p* = .034). Grade, nonverbal IQ, and English receptive vocabulary all made contributions to English reading (all *ps* < .001). Other predictors did not contribute significantly (*p*s > .05).

We further reported a multiple linear regression model where all the predictors were added to the model. English VSL, non-linguistic ASL, Chinese ASL, PPVT, Raven, and grade were all significant predictors (all *ps* < .05). Other predictors did not contribute significantly (*p*s > .05), see Table 4.

**Table 4:** Summary of multiple linear regression analysis for variables predicting performance on English word reading efficiency.

Variables	*ß*	*SE*	*t*
grade	0.35	0.62	6.42***
English vocabulary	0.30	0.32	5.25***
Non-verbal IQ	0.19	0.23	3.68***
NVSL	0.05	7.04	0.87
NASL	0.16	8.78	2.70**
EVSL	0.18	6.93	3.37**
EASL	-0.10	8.67	-1.76
CVSL	-0.02	7.22	-0.33
CASL	0.12	7.11	2.14*

### Predicting Chinese word reading

To examine the unique contribution of SL in explaining individual variances in Chinese word reading efficiency, we ran the hierarchical linear regression model. Grade was center coded, and we used raw scores for the reading measures. Chinese word reading efficiency was entered as the outcome variable (See Table 5).

**Table 5:** Summary of hierarchical regression analysis for variables predicting performance on Chinese word reading efficiency.

Variables	*ß*	*SE*	*t*	*R*	*R* ^*2*^	Δ*R*^*2*^
**Step 1**				0.51	0.26	0.26
grade	0.32	0.99	3.56***			
Non-verbal IQ	0.28	0.38	3.16**			
**Step 2**				0.57	0.32	0.06
NVSL	-0.92	11.34	-0.08			
NASL	0.29	13.97	3.06**			
**Step 3**				0.60	0.36	0.04
CVSL	0.22	12.06	2.38*			
CASL	0.25	11.9	0.20			
**Step 4**				0.64	0.41	0.05
EVSL	-0.23	11.50	-2.50*			
EASL	0.21	14.27	2.15*			

*Note*. NVSL = Non-linguistic VSL; NASL = Non-linguistic ASL; EVSL = English VSL; EASL = English ASL; CVSL = Chinese VSL; CASL = Chinese ASL.

*p < .05

**p < .01

***p < .001.

In the regression model, grade and nonverbal IQ were entered as a block as control factors in the first step. In the second step, we entered non-linguistic VSL and ASL as a block to examine how non-linguistic SL contributes to Chinese word reading efficiency. In the third step, we entered Chinese VSL and ASL as a block to examine how Chinese SL contributes to Chinese reading independent of non-linguistic SL. In the final step, we entered English VSL and ASL as a block to examine how English SL makes a cross-linguistic contribution to Chinese word reading efficiency beyond non-linguistic SL and Chinese SL.

Non-linguistic ASL made a unique contribution after controlling grade and nonverbal IQ (*β* = .29, *SE* = 13.98, *t* = 3.06, *p* = .003). Chinese VSL contributed a significant amount of variance beyond non-linguistic SL (*β* = .22, *SE* = 12.07, *t* = 2.38, *p* = .019). Importantly, English VSL and ASL both made unique contributions beyond Chinese SL. However, English VSL seemed to be negatively associated with Chinese reading (English VSL: *β* = -0.23, *SE* = 11.50, *t* = -2.50, *p* = .014; English ASL: *β* = 0.21, *SE* = 14.27, *t* = 2.15, *p* = .034). Grade and non-verbal IQ both made significant contributions to Chinese reading (*ps* < .05), whereas non-linguistic VSL and Chinese ASL did not (*p*s > .05).

We also reported a multiple linear regression model where all the predictors were added to the model. English VSL, Chinese VSL, non-linguistic ASL, English ASL, and grade were all significant predictors (all *ps* < .05). Other predictors did not contribute significantly (*p*s > .05), see Table 6.

**Table 6:** Summary of multiple linear regression analysis for variables predicting performance on Chinese word reading efficiency.

Variables	*ß*	*SE*	*t*
grade	0.24	1.02	2.64**
Non-verbal IQ	0.17	0.38	1.98
NVSL	-0.09	11.69	-0.84
NASL	0.23	14.51	2.31*
CVSL	0.20	12.00	2.15*
CASL	0.07	11.83	0.69
EVSL	-0.23	11.50	-2.50*
EASL	0.21	14.27	2.15*

## Discussion

The goal of the present study was to examine whether and how non-linguistic SL, Chinese SL, and English SL contribute to Chinese L1 word reading efficiency and English L2 word reading efficiency among a group of native Chinese-speaking fourth, sixth, and eighth graders who learn English as L2. Several important findings were obtained from this study. First, children showed above-chance learning across all the SL tasks including non-linguistic, Chinese and English, and across all grades. In general, there were developmental improvements across at least two of the three grades on all SL tasks. Second, non-linguistic ASL, English VSL, and Chinese ASL accounted for a significant amount of variance in English word reading efficiency. Third, non-linguistic ASL, Chinese VSL, English VSL, and English ASL all accounted for a significant amount of variance in Chinese word reading efficiency. Finally, grade and non-verbal IQ both contributed to English and Chinese word reading efficiency. English receptive vocabulary contributed to English word reading efficiency.

### Contribution of SL to reading

Our data provide novel evidence about cross-linguistic contribution both from Chinese SL to English reading and from English SL to Chinese reading. Consistent with our hypothesis, we showed that Chinese ASL predicted a significant unique amount of variance in English reading over and above non-linguistic SL, English SL, and control measures. Children tapped into Chinese ASL for English word reading even though their English ASL did not contribute. It seems that children who are familiar with and skilled at their Chinese L1 are more prone to tap into their L1 ASL to support L2 reading. Nevertheless, deviant from the original hypothesis, our results showed both English VSL and ASL were uniquely associated with Chinese word reading over and above all other measures.

In line with previous research that L2 experiences can influence L1 reading skills, resulting in either facilitation or inference depending on the properties of the L2 writing system (e.g., [51]), our results suggest that children are able to tap into their L2 SL in L1 reading. However, one may ask why English ASL facilitated Chinese word reading whereas English VSL showed interference. We speculate that English ASL and Chinese word reading may share some underlying processes such as the composition of syllables to form multisyllabic words. Specifically, each English ASL triplet is formed by combining three open syllables, following a “CVCVCV” structure, which may connect to how Chinese open syllables are combined to form Chinese words. The interfering role of English VSL in Chinese word reading may be explained by the sharp contrast between Chinese and English visual writing systems, such that English VSL may interfere with Chinese word reading. Taken together, there seems to be a bi-directional cross-linguistic influence between linguistic SL and reading across different writing systems, either facilitating or interfering.

Previous research suggests that non-linguistic SL plays a role in learning to read deep orthography like English, semi-transparent orthography like Norwegian and transparent orthography like Spanish (e.g., [20, 23, 52]). The present study provides not only converging evidence for the role of non-linguistic SL in learning to read in English, consistent with [21, 53], but also novel evidence that non-linguistic SL is related to learning to read in Chinese, a non-alphabetic writing system. Specifically, our data revealed that non-linguistic ASL contributed significantly to both English and Chinese reading. We suggest that better perceptual sensitivity to acoustic features as well as distributional patterns embedded in the auditory inputs in non-linguistic ASL contributes to sensitivity to phonological information in speech (e.g., [54, 55]), which may be associated with phonological awareness essential for learning to read across different writing systems (e.g., [56]). The non-linguistic VSL, on the other hand, is unlikely to contribute to phonological awareness, and the alien images are also unlikely to contribute to the visual processing of the written words. The nature of the specific visual materials used in the current non-linguistic VSL might help explain their nonsignificant contribution to reading in either English or Chinese.

Our study also observed a unique contribution of Chinese VSL to Chinese word reading. Notably, previous research on Chinese SL mostly focused on children’s sensitivity to regularities within characters (e.g., [2, 31]). Our study provides novel evidence that sensitivity to the statistical patterns embedded in a sequence of Chinese characters contributes to reading at the word level. Within English, independent of non-linguistic SL, English VSL predicted English word reading, whereas English ASL did not. This may be explained by the status of English as L2 by native Chinese-speaking children. Native Chinese-speaking children rely more on visual-orthographic compared to phonological information in processing English words (e.g., [57]). This processing strategy may lead to children’s better detection of visual regularities in English written stimuli and result in more efficient reading of English words.

### Domain-general vs. domain-specific SL

The findings of our study may differ from the idea that SL operates generally across different domains. Our research indicates that only auditory SL, not visual SL, is associated with reading proficiency in both English and Chinese. Furthermore, our results suggest that visual SL is the only predictor of reading proficiency within each language, while Chinese auditory SL predicts English reading and both English auditory and visual SL predict Chinese reading in the cross-language prediction. Based on these findings, it seems less likely that there is a single learning system that underlies all types of SL. It is possible that there is a robust SL mechanism but with the possibility of peripheral differences.

In conclusion, our study supports the idea of the multi-componential theory of SL ([12–14, 27, 32]). This theory suggests that SL cannot be considered as a single construct, and different SL tasks may access different sub-components of SL that are not interchangeable, or may access the same sub-component with varying weights. For instance, working memory is likely to be underlying sub-component of SL, and some SL tasks in our study may have required more working memory than others. Working memory has a significant impact on reading efficiency, which may explain why some SL tasks predicted reading efficiency more significantly than others (e.g., [58, 59]). The componential view of SL may help to explain this phenomenon. However, we were not able to distinguish the specific components underlying SL in our study design. Future research may incorporate tasks that directly measure specific components such as working memory or use neuroimaging techniques to observe how SL operates with the support of different brain regions. While research on the nature of SL is ongoing, our study highlights the importance of examining SL in various modalities and domains when exploring individual differences between SL and reading.

### Limitations, future directions, and educational implications

There are several limitations in our study. First, given that our study employed a cross-sectional design, it was unable to capture how the relationship between SL and reading may arise and change over the course of reading development. A longitudinal design would be extremely valuable for investigating the developmental trajectory and a potential causal relationship between SL and reading. Another limitation is that our design did not include a measure of phonological awareness, a well-established predictor of reading outcomes in both alphabetic and non-alphabetic orthographies [60, 61]. It would be worthwhile to examine the role of phonological awareness in the relationships between SL and reading in both Chinese L1 and English L2. Third, the present study only measured word-level reading. It would be interesting for future research to take sentence-level and passage-level reading into consideration and see if the pattern is similar across different reading materials. Fourth, our study only focused on one type of SL related to transitional regularities. Other types of statistical patterns such as distributional regularities also occur across languages (e.g., [14]). Caution is needed in generalizing the current results to other types of SL. Finally, future research may consider incorporating a pseudo-word reading task to more precisely investigate the role of visual-orthographic and phonological factors in cross-linguistic reading processes. Pseudo-words, which require a more phonological approach to reading instead of a direct route, could provide a clearer contrast between the reliance on visual-orthographic and phonological information in reading Chinese versus English.

Our findings have several educational implications. First, our result that SL is related to literacy skills may bring important implications for designing instructional materials in the classroom. Teachers may integrate an SL component in their literacy curriculum in both visual and auditory modalities and in both linguistic and non-linguistic domains. Early literacy intervention programs could pay attention to designing appropriate reading materials to help develop children’s sensitivity to the statistical information in their writing system. Apfelbaum et al. [62] for example, demonstrated that if the training materials are designed to involve certain grapheme-to-phoneme relations (e.g., the short vowel “a” in “fat” is pronounced as “æ”), children as young as first grade could learn and generalize these regularities. Moreover, based on our survey, most teachers rarely emphasize the importance of SL patterns in teaching English L2. Our results suggest that teachers may pay more attention to statistical patterns embedded in L2 in their teaching (e.g., the spelling rule of double consonants in English and some particular word endings in English may cue the position of lexical stress in English) in both spoken and written words and children’s improved sensitivity to these patterns is likely to predict improvement in their reading skills. Finally, we revealed that detecting statistical regularities in one language can contribute to successful reading in another language, which brings implications for students struggling with L2 reading. That is, educators can develop SL practices and intervention programs in L1 as a pathway to improve students’ L2 reading.

## Conclusions

The present study systematically examined the contribution of non-linguistic and linguistic SL to Chinese L1 and English L2 word reading. Our results reveal that SL in both linguistic and non-linguistic domains was significantly associated with Chinese and English word reading. More importantly, we provide clear evidence for cross-linguistic contribution from Chinese SL to English reading, and from English SL to Chinese reading, highlighting a bi-directional relationship between SL in one language and reading in another.

## Supporting information

S1 Text Development of statistical learning across modalities, domains, and languages. (PDF)
