# Supplementary material for: Contribution of statistical learning in learning to read across languages
Source: PLoS One. 2024 Mar 25;19(3):e0298670. doi: 10.1371/journal.pone.0298670 (PMC10962809; doi:10.1371/journal.pone.0298670)
Supplement: S1 Text — (PDF) [file pone.0298670.s001.pdf]

Journal of Experimental Child Psychology 226 (2023) 105570

Contents lists available at ScienceDirect

Journal of Experimental Child Psychology

journal homepage: www.elsevier.com/locate/jecp

# Development of statistical learning ability across modalities, domains, and languages

Jinglei Ren, Min Wang *

*Department of Human Development and Quantitative Methodology, University of Maryland, College Park, College Park, MD 20742, USA*

ARTICLE INFO

*Article history:*
Received 6 February 2022
Revised 22 September 2022

*Keywords:*
Statistical learning
Development
Modality
Domain
Language

ABSTRACT

Statistical learning (SL) is defined as our ability to use statistics (e.g., frequencies or transitional probabilities) to detect implicit regularities in the environment. Limited research has examined the developmental trajectory of SL across domains and modalities, and no previous research has made systematic comparisons across domains, modalities, and languages using comparable tasks. The current study investigated the development of SL ability across 9-, 11-, and 13-year-old native Chinese-speaking children in non-linguistic visual and auditory SL, first-language Chinese visual and auditory SL, and second-language English visual and auditory SL. Results showed that children across the three age groups achieved all types of SL, and they performed better in visual modality than in auditory modality. Furthermore, while visual SL constantly improved from 9- to 11- to 13-year-olds, auditory SL improved only from 11- to 13-year-olds but not from 9- to 11-year-olds, which could be explained by the discrepancy in developmental trajectory between auditory language and working memory. This pattern of age and modality interaction was similar across non-linguistic Chinese and English SL. A significant interaction between modality and language type also showed that better learning was achieved in visual SL as compared with auditory SL in both non-linguistic and English stimuli. However, children performed similarly across the two modalities in Chinese, possibly due to the contribution of tonal information. Together, our findings point to the joint function of age, modality, and language type in SL development.

© 2022 Elsevier Inc. All rights reserved.

* Corresponding author.
*E-mail address:* minwang@umd.edu (M. Wang).

https://doi.org/10.1016/j.jecp.2022.105570
0022-0965/© 2022 Elsevier Inc. All rights reserved.

## Introduction

Humans are sensitive to regularities in environment. One prominent proposed mechanism underlying this sensitivity is statistical learning (SL). In the current study, we defined *statistical learning* as the ability to use statistics (e.g., transitional probabilities or frequencies) to discover boundaries of sequentially presented stimuli. SL is present from infancy and is robustly observed across different modalities (e.g., visual, auditory, tactile) during the first year of life (e.g., Emberson et al., 2020; Kirkham et al., 2002; Saffran et al., 1996) and continues to develop throughout childhood, at least in the visual modality (e.g., Arciuli & Simpson, 2011; Raviv & Arnon, 2018). Previous research also investigated the differences in SL across linguistic and non-linguistic stimuli. For instance, the classic work by Saffran et al. (1996) showed that both infants and adults were able to detect statistical regularities in non-linguistic sequences of "tone words" and linguistic sequences of English syllables. In terms of development, some studies argued that SL is an early-maturing capacity that is not expected to improve with age (e.g., Bertels et al., 2015; Saffran et al., 1997), whereas many other studies showed a clear developmental trend in SL (e.g., Arciuli & Simpson, 2011; Shufaniya & Arnon, 2018). Recent research suggests that the development of SL is modality dependent and domain dependent (e.g., Raviv & Arnon, 2018; Shufaniya & Arnon, 2018).

The current study systematically examined development of SL ability across 9-, 11-, and 13-year-olds in non-linguistic visual and auditory SL, first-language (L1) Chinese visual and auditory SL, and second-language (L2) English visual and auditory SL. We focused on the SL in Chinese L1 and English L2 given their contrasting writing systems (non-alphabetic vs alphabetic) characterized by qualitatively different statistical properties. Limited research has examined Chinese SL in the visual modality (see He & Tong, 2017, and Yin & McBride, 2015, for exceptions) and in the auditory modality (see Wang & Saffran, 2014, for an exception). However, no research has systematically studied the developmental trajectory of Chinese SL in comparison with English SL via comparable tasks. Making comparisons in SL across Chinese and English contributes to our understanding of SL beyond the English language and writing system. The findings from the current study shed new light on our better understanding of the development of SL across modalities, domains, and writing systems during late childhood.

### *SL during early infancy*

Considering the richness and complexity of a natural language, why and how can infants learn words so rapidly, especially when speakers do not mark word boundaries with pauses? Saffran et al. (1996) proposed a mechanism for statistical word segmentation—transitional probability (TP) detection. *Transitional probability* refers to the probability of one syllable occurring given the previous syllable (Enochson, 2015).[1] Syllables within a word have a higher TP than syllables across word boundaries. For example, in the sequence *pretty baby* the likelihood of the syllable *pre* being followed by the syllable *ty* is high, whereas the probability of the syllable *ty* in *pretty* being followed by the syllable *ba* in *baby* is low. Segmenting the two words using TP would result in a word boundary correctly located between the sequence *pretty* and the sequence *baby.*

Saffran et al. (1996) presented 8-month-olds with a synthesized speech stream consisting of four three-syllable "words" composed of 12 unique syllables (e.g., tupiro, golabu, dapiku, and tilado) in a random order (e.g., dapikutupirotilado-golabutupiro . . .) for 2 min. The only cues to word boundaries were the TPs between syllable pairs. For example, the TP of *tu-pi* in this corpus was high (1.00) because *pi* always followed *tu* within the word *tupiro* (a within-word syllable pair), whereas the TP of *ro-go* was low (0.33) because *golabu* was one of three words that could follow *tupiro* (a between-word syllable pair). After brief exposure to this language, infants were able to distinguish between high-TP syllable sequences and low-TP sequences, suggesting that infants are sensitive to the TP information contained in the speech stream and sensitivity to TPs is a possible mechanism underlying language acquisition (Saffran et al., 1996).

[1] The formula for transitional probability is $TP(A \rightarrow B) = \frac{\Pr(AB)}{\Pr(A)}$. A high TP indicates that B is likely given A, whereas a low TP indicates that B is unlikely given A.

One may wonder the following: Is SL only a property of language acquisition, or could it also apply to non-linguistic stimuli? Saffran et al. 1999), modeled directly after Saffran et al. (1996), examined infants' performance on tone stimuli. The tones were combined into triplets to form six "tone words" (ADB, DFE, GG#A, FCF#, D#ED, and CC#D). These tone words were created by translating the previous nonsense words (e.g., bu became the musical note D, bupada became DFE). A two-alternative forced-choice (2AFC) task was employed to assess learning, similar to the task in Saffran et al. (1996). In the test phase, each test item consisted of two tone sequences: a word and a non-word (e.g., ADB and AFG#). Non-words consisted of three-tone sequences made of tones drawn from the previous tone sequences but had never occurred in that order during exposure. Results showed that infants were able to detect statistical regularities in non-linguistic sequences of tone words, suggesting that statistical structure can be extracted from auditory input regardless of the domain in which it is presented (syllables or tones).

The aforementioned research has revealed powerful SL ability in infants given that they have the capacity to extract statistical regularities from a variety of linguistic and non-linguistic auditory input. In fact, SL during early infancy not only is limited to auditory information but also extends to visual modality. A representative study is Kirkham et al. (2002), where 2-, 5-, and 8-month-old infants were habituated to sequences of discrete visual stimuli whose ordering followed a statistically predictable pattern (e.g., Pair 1: turquoise square followed by blue cross; Pair 2: yellow circle followed by pink diamond; Pair 3: green triangle followed by red octagon). The infants subsequently viewed the familiar pattern alternating with a novel sequence of identical stimulus components and exhibited significantly greater interest in the novel sequence at all ages, suggesting that there is a modality-general SL that is present among very young infants. Researchers also investigated the developmental trajectory of SL across modalities in infants. For example, Emberson et al. (2020) compared visual and auditory SL in 8- to 10-month-old infants and showed that auditory SL increased, whereas visual SL did not for this age range.

In sum, there has been a great deal of evidence that SL is present from early infancy (e.g., Saffran et al., 1996; Teinonen et al., 2009) and is shown across different modalities and domains (e.g., Kirkham et al., 2002; Emberson et al., 2020; Saffran et al., 1999; see Saffran & Kirkham, 2018, for a review). However, relatively fewer studies have examined SL during childhood, and limited research that addressed the developmental trajectory of SL showed a mixed pattern of results.

*Development of SL across modalities and domains*

One of the key questions in this line of research is whether SL is age invariant (e.g., Saffran et al., 1997) or can be improved with age (e.g., Arciuli & Simpson, 2011). Evidence thus far suggests that the development of SL is largely dependent on modalities or domains (e.g., Raviv & Arnon, 2018; Shufaniya & Arnon, 2018). Auditory SL has been considered as an early-maturing capacity that is not expected to improve with age (e.g., Saffran et al., 1997). In the non-linguistic domain, Saffran et al. (1997) exposed both 6-year-olds and undergraduate students to an unsegmented artificial language, presented auditorily, in which the only cues to word boundaries were the TPs between syllables. No significant difference in auditory SL was shown between 6-year-olds and undergraduate students. Qi et al. (2019) employed the same paradigm and found that children and adults performed similarly. In the linguistic domain, Raviv and Arnon (2018) examined linguistic auditory SL in children aged 5 to 12 years and showed that linguistic auditory SL did not improve with age, consistent with Saffran et al. (1997). However, Shufaniya and Arnon (2018) argued that the linguistic nature of the stimuli (syllables), rather than its auditory modality, was responsible for the lack of improvement with age in previous work. The development of SL is dependent on the domain (linguistic vs non-linguistic stimuli) rather than the modalities. Children aged 5 to 12 were tested using a series of non-linguistic auditory stimuli (familiar sounds, e.g., door opening, bell ringing), and a clear developmental trend of improvement in auditory SL from 5 to 12 years was shown.

Regarding visual SL, evidence suggests that it matures at about 10 years of age. Bertels et al. (2015) reported that children (9–12 years) performed similarly to adults on a shape (e.g., arrow) visual SL task. Likewise, Schlichting et al. (2017) showed no significant difference between children (6–11 years) and adolescents (12–17 years) on a similar visual SL task. However, there is also evidence for a clear

developmental trend in visual SL. Arciuli and Simpson (2011) examined visual SL in children aged 5 to 12 years in which 12 aliens were divided into four groups of three (four base triplets) referred to as ABC, DEF, GHI, and JKL. The familiarization phase consisted of a continuous stream of aliens displayed on a computer screen one at a time. For each test trial, one base triplet was displayed together with one impossible triplet, and participants were prompted to identify which of the two triplets had appeared previously (during familiarization). A significant effect of age was shown, with older children performing much better than younger children. In addition, Raviv and Arnon (2018) and Shufaniya and Arnon (2018) used familiar objects (e.g., house, book, plane) in their visual SL tasks. Both studies showed improvement during childhood from 5 to 12 years of age. Note that all these studies examined visual SL using non-linguistic visual stimuli (e.g., shapes, aliens, objects), with very limited research investigating linguistic visual SL (e.g., syllables, characters) using the standardized SL paradigm. Isbilen et al. (2020) employed a statistically induced chunking recall task to tap into SL among adults of visually presented English syllables. However, no previous work has examined the developmental trend of linguistic visual SL during childhood. The current study filled this gap by examining the linguistic visual SL in both English and Chinese.

The current study aimed to systematically examine SL development across ages, domains (linguistic vs non-linguistic), modalities (visual vs auditory), and languages (Chinese vs English). Most previous research focused on only a single modality (e.g., auditory: Saffran et al., 1997; visual: Arciuli & Simpson, 2011). Broadbent et al. (2018); Broadbent, White, Mareschal and Kirkham (2017) examined the role of task modality in incidental category learning in 6- to 10-year-olds. Their results revealed that engaging in an auditory concurrent task led to poorer performance on incidental category learning compared with an audiovisual or visual concurrent task. Raviv and Arnon (2018) and Shufaniya and Arnon (2018) compared the developmental trajectory of non-linguistic visual, non-linguistic auditory, and linguistic auditory SL, but the development of linguistic visual SL (e.g., English syllables) was not examined in their two studies. Moreover, compared with the aforementioned two studies that recruited different participant samples in comparing different SL types, our study had the same participants complete a set of different types of SL tasks for a more direct comparison across modality. Finally, whereas most previous studies used linguistic stimuli in one language with a heavy focus on English, we compared the developmental pattern of SL in two different languages (Chinese L1 and English L2).

In summary, the current study investigated developmental changes in SL across visual and auditory modalities, across linguistic and non-linguistic domains, and across Chinese and English writing systems simultaneously with comparable experimental tasks within the same sample of participants. Findings contribute to our better understanding of whether and how SL develops, like many other cognitive capacities, and the contribution of modality, domain, and language.

### *SL in the Chinese writing system*

The Chinese writing system provides a fascinating window into the visual SL mechanisms due to its unique orthography and statistical consistencies. Unlike English and other alphabetic orthographies, the basic orthographic unit in Chinese is a character that maps onto a syllable and a morpheme. A Chinese spoken syllable consists of an optional initial consonant, a vowel (accompanied by tone), and an optional final nasal consonant (n or ng). There are four tones in Chinese syllables: level (Tone 1, ā), rising (Tone 2, á), falling–rising (Tone 3, ǎ), and falling (Tone 4, à). For example, the syllable segment *ma* accompanied by four different tones carries four different meanings: *mā* 'mother', *má* 'hemp', *mǎ* 'horse', and *mà* 'scold' (Chao, 1968). Most Chinese characters (>80 %) are semantic–phonetic compounds containing a semantic radical that provides a cue to the meaning of the whole character and a phonetic radical that provides a cue to the pronunciation of the whole character (Shu et al., 2003). For example, the character 枝 (*branch*) /tʂɨ1/ consists of a semantic radical 木(*wood*) indicating a *wood*-related concept and a phonetic radical 支/tʂɨ1/ providing a cue to the pronunciation of the whole character. Moreover, radicals exhibit certain positional consistencies; semantic radicals are usually on the left or top position and phonetic radicals are on the right or bottom position in the left–right and top–bottom structured characters, respectively (e.g., Shu et al., 2003).

Beyond the character level, most Chinese vocabularies are compound words that consist of two or three characters (Shu & Anderson, 1997). For example, the Chinese word for 'train' is 火车 (huǒ chē), which literally means "*fire car.*" Likewise, a helicopter—直升机 (zhí shēng jī)—is a "*straight-rising machine.*" There are three types of typical Chinese compounds: subordinate, attributive, and coordinated (Ceccagno & Basciano, 2007). In subordinate compounds, one word clearly subordinates (or supports) another; for example, 房型 (fáng xíng) means "*layout of house*" (house + model). In attributive compounds, the descriptive character precedes the noun/verb; for example, 互动 (hù dòng) means "*interaction*" (mutual + to move). In coordinated compounds, all the characters are equally important in determining the meaning; for example, 高矮 (gāo'ǎi) means "*height*" (high + low). Therefore, the sequential patterns between characters are useful information in determining the meanings of Chinese words.

Previous research investigated children's SL of positional consistencies within individual Chinese characters. For example, the corpus analysis of Chinese characters revealed that the consistency with which 木appears on the left of left–right characters is 64.5 %. Native Chinese-speaking children can extract statistical regularities from Chinese visual stimuli starting at a young age. Yin and McBride (2015) showed that native Chinese-speaking children are sensitive to visual–orthographic regularities before they receive formal literacy instruction in Chinese. In a character learning task, 4-year-old children performed better when phonetic cues were available compared with when the cues were absent, and 5-year-olds performed better when radicals in the stimuli were positioned legally than when they were positioned illegally. Such sensitivities demonstrate that native Chinese kindergarteners can detect statistical patterns in Chinese characters without being taught explicitly. Furthermore, He and Tong (2017) employed a modified classical SL paradigm (i.e., artificial orthography learning) in which a set of Chinese-like logographic characters was created using an ideographic script (Dongba) and a syllabic script (Geba). After a short exposure to a subset of novel logographic characters, children in Grades 3 to 5 (~8–10 years of age) were able to distinguish characters containing radicals in legal positions from those in illegal positions. Moreover, children with developmental dyslexia were less sensitive to the statistical regularities (i.e., positional consistencies of radicals) compared with normal-achieving learners (Lee & Tong, 2020). Clearly, the previous research related to SL in Chinese has focused on within-character statistical patterns. The current study focused on sequential patterns in combining characters at the Chinese word level. We employed character triplets similar to English triplets in terms of the combination of written syllables to tap into children's sensitivity to between-character sequential regularities.

## *The current study*

We examined the development of SL using comparable tasks across domains and modalities in Chinese L1 children aged 9, 11, and 13 years who learn English L2. There are two primary reasons for choosing this age range. First, we were interested in comparing our results with previous studies; therefore, we decided on this age range that is within the most studied in the previous work (5–12 years) (Arciuli & Simpson, 2011; Raviv & Arnon, 2018; Saffran et al., 1997; Shufaniya & Arnon, 2018). Second, because we included L2 English linguistic SL tasks, we wanted to make sure that our participants were able to complete the L2 tasks. By the age of 9 years, children in China have received 1 year of formal English instruction and so were a good starting point to be included in the current study.

There were three primary research questions guiding this study. First, does SL ability improve across ages? Second, does the development of SL differ across domains (non-linguistic vs linguistic) and across modalities (visual vs auditory)? Third, is the developmental trajectory different in SL between Chinese L1 and English L2? For the first two research questions, based on previous evidence that both non-linguistic visual and non-linguistic auditory SL improved with age, but the linguistic nature of auditory SL stimuli resulted in age invariance given that linguistic knowledge of auditory stimuli is expected to be well established during early childhood (e.g., Raviv & Arnon, 2018; Shufaniya & Arnon, 2018), we hypothesized that the developmental trajectory for non-linguistic and linguistic SL would be different in that both non-linguistic visual and non-linguistic auditory SL

would improve with age. For linguistic SL, on the other hand, only visual would improve with age and auditory would not in both Chinese and English.

For the third research question, given that Chinese characters contain salient visual features, Chinese children would rely more on visual processing in reading Chinese compared with auditory processing (e.g., Yang & Meng, 2020). Furthermore, Chinese children may adopt their visual processing strategies in Chinese to read and process English words (e.g., Wang & Geva, 2003). Hence, we hypothesized that there may be a modality-based advantage in both English and Chinese; that is, children would perform better in English visual than in English auditory and would perform better in Chinese visual than in Chinese auditory. In addition, within Chinese visual SL, we were interested in whether the specific Chinese orthographic structure (top–bottom vs left–right) plays a role. Tong and McBride (2014) suggested that Chinese top–bottom structure is more predictable than left–right structure; thus, we hypothesized that our participants were likely to perform better on the visual SL of top–bottom structure. We were also interested in examining the correlation between L2 proficiency and L2 SL. We hypothesized that there should be a significant correlation between L2 proficiency and SL in L2. The higher the L2 vocabulary is, the better the sensitivity is to the statistical patterns of the L2 constituent syllables, which then could be linked to better SL with both auditory and visual stimuli.

Following the standardized SL triplet paradigm (Arciuli & Simpson, 2011), we designed six SL tasks in the current study. Specifically, there were two SL tasks in the non-linguistic domain: a non-linguistic visual and a non-linguistic auditory. The well-established triplet paradigm included a familiarization phase and a forced-choice test phase. The familiarization phase consisted of four triplets, displayed as a continuous stream of aliens, shown one at a time. There were four new triplets created in the test phase, and participants were asked to identify the triplet they saw previously. The non-linguistic visual stimuli were adapted from Arciuli and Simpson (2011), and 12 alien images were created to form four base triplets. The non-linguistic auditory stimuli were modeled after Qi et al. (2019) and included 12 pure tones divided into four groups. In addition, four tasks in the linguistic domain—English visual and auditory and Chinese visual and auditory, comparable to the non-linguistic tasks—were administered. The English visual task was modeled after Kidd et al. (2020), in which four triplets—kibudu, modipa, takapo, and lomari—were employed. The English auditory task was adapted based on Schneider et al. (2020) and consisted of four English syllable triplets. Children in the current study were familiar with syllables in English visual SL and auditory SL, which were included in their English textbooks and taught in the classroom. The Chinese visual task stimuli were half borrowed from He and Tong (2017) and half located via an online source (https://www.pinterest.ca/pin/491455378072678746). Twelve pseudo-Chinese characters were divided into four base triplets. These characters represent Chinese basic orthographic structures, half top–bottom and half left–right. We also created Chinese auditory stimuli to be comparable to the English auditory stimuli. Chinese tones were incorporated into the stimuli to reflect the Chinese tonal feature. Each syllable in a triplet was assigned a tone out of the four basic Mandarin tones, and three syllables within the triplet were ensured to carry different tones. In addition, children received an English receptive vocabulary test as an index of their English L2 proficiency.

## Method

### Participants

We recruited children aged 9 years ($M$ = 9.2 years; 17 male), 11 years ($M$ = 11.1 years; 15 male), and 13 years ($M$ = 13.5 years; 18 male), 40 in each age group, who were from a mid-sized city in northern China. All the participants were native Chinese speakers and had at least 1 year of formal English instruction. We recruited students whose scores on their English exams in the past academic year were among the top 50 % in the class. Parental consent was obtained. Parents were asked to fill out a questionnaire for information about their children's home language exposure and experiences. Most parents (76 %) had a high school diploma, whereas the remaining parents had a college degree. Regarding English proficiency, 22 % of the parents had CET-4 (College English Test–Level 4, a common

standard test assessing English proficiency for college students in China), whereas 78 % had only high school English proficiency or lower. Among the children, 87 % started to learn English in Grade 3, 7 % started from Grade 2 onward, and 6 % started from Grade 1 onward. Fully 82 % only spoke English in their English classes at school, and 18 % received private lessons taught by non-native English speakers ranging from 2 to 5 h (average = 3.75 h) per week outside school. All these children never spoke English at home.

We also asked three teachers who taught English to our participants to fill out a questionnaire regarding their teaching approach employed in English class instruction. All these teachers applied *whole-language-based* instruction, which means that the curriculum does not include any phonics instruction. Two of them had English proficiency at the CET-4 level, whereas the other one had CET-6 level. Each English class lasted 45 min, and children received five lessons each week. Reading, writing, and vocabulary were ranked as the most important tasks by the three teachers for their instruction. Speaking and listening were ranked as less important. One teacher reported that she sometimes emphasized the importance of specific spelling patterns such as double consonants and plural suffixes, and the other two did not emphasize any spelling patterns.

All the following SL tasks were composed of a familiarization phase and a testing phase. Children either saw (in visual SL tasks) or heard (in auditory SL tasks) a sequence of stimuli in the familiarization phase. There were 32 2AFC trials in each SL test phase. The experimenter provided verbal instructions to the children throughout the two phases and monitored to make sure that children stayed on-task. There was no time constraint for responses and no feedback on accuracy of answers in the test phase. All the SL tasks were written in *jsPsych* (de Leeuw, 2015) and run in by an R package named *jsPsychR* in R Studio (RStudio Team, 2020).

Ethics approval was obtained from the institutional review board at the University of Maryland, College Park. Informed consent was obtained from parents.

### *Non-linguistic visual SL*

Modeled after Arciuli and Simpson (2011), 12 unique alien images were divided into four groups of 3 images to create four base triplets (ABC, DEF, GHI, and JKL). For the test phase, four foil triplets were created. The relative position of each image in a foil triplet was the same as the base triplets, but the images were grouped into four foil triplets referred to AEI, DHL, GKC, and JBF.

Children saw and heard at the beginning, “Hi there, today you are going to see some aliens line up to enter a cool spaceship. We need you to help us keep track of a very special alien as the aliens line up to enter their spaceship. We will show you the alien now.” In the familiarization phase, we repeated each of the four base triplets 24 times for a total of 96 triplets. Aliens were presented one at a time on a computer screen for 800 ms each with a 200-ms interstimulus interval. In the familiarization phase, children were instructed to press the spacebar as quickly as possible whenever the specific target alien appeared on the screen. The target alien image was always the third alien of one of the four base triplets (see upper part of Fig. 1 for the example of image presentation order). The 2AFC test phase was introduced after the familiarization phase. At the beginning of the test phase, children heard and saw, “Now you are going to see two groups of aliens, three as a group, and one of the two groups was friends! The friend group always followed each other to enter the spaceship. Please select the group that you think they were friends before.” Participants were asked to identify which of the two triplets (one was the base triplets and the other was the foil) seemed more like what they saw in the familiarization phase.

### *Chinese visual SL*

Modeled after He and Tong (2017), 12 pseudo-characters were created by combining two Geba characters (e.g., 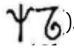). Because some Geba characters originate from Chinese loan characters, they are visually similar to Chinese characters (Zhou, 2014). Geba characters have the same structure (e.g., top–bottom, left–right) as real Chinese characters and share many similar strokes with the mod-

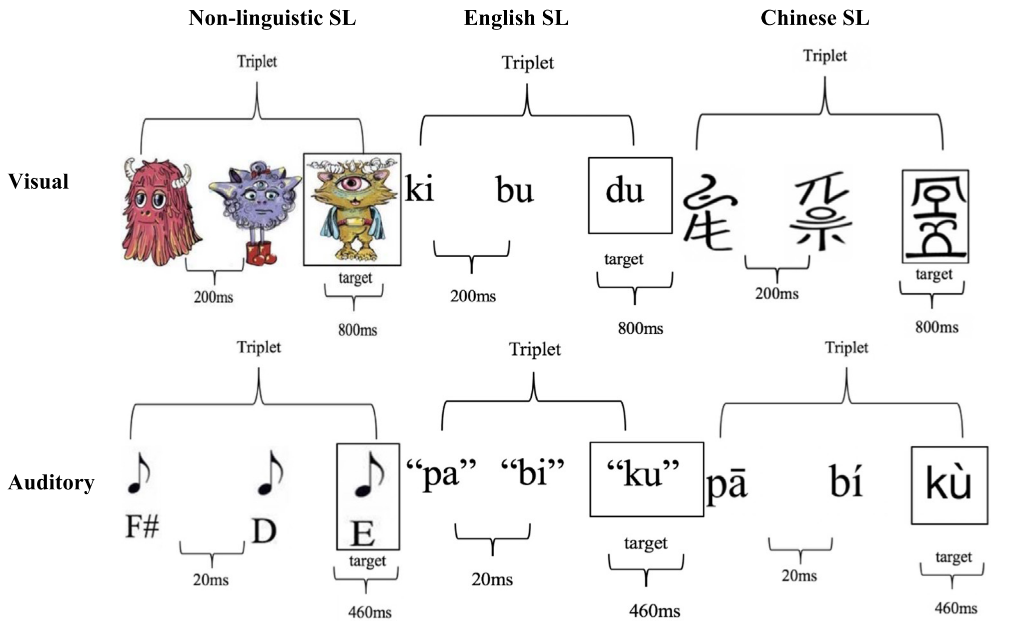


**Fig. 1.** Familiarization phase of all six statistical learning (SL) tasks. Example triplets across each task are depicted in this figure. Each visual stimulus appeared for 800 ms with a 200-ms interval, and each auditory stimulus was heard for 460 ms with a 20-ms interval.

ern Chinese characters. In addition, they are meaningless, thereby offering a unique tool for testing children’s implicit learning of sequential patterns of written Chinese. There were 6 left–right pseudo-characters (e.g., 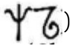) and 6 top–bottom pseudo-characters (e.g., 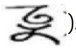), reflecting the two commonly used character structures in Chinese (Shu et al., 2003). These 12 pseudo-characters were divided into four base triplets, for example, 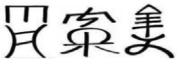. See Table 1 for the full set of stimuli. These characters were visually presented in a sequence on the screen.

The Chinese visual SL procedure was the same as the non-linguistic visual SL procedure. Children heard and saw at the beginning, “Hi there, we are going to learn a new language today! We need you to help us keep track of a special character as you see different characters appearing on the screen. We will show you the characters now.” At the beginning of the test phase, children heard and saw, “Now you are going to see two groups of characters, three as a group. Please select the group of characters that always followed each other appearing on the screen before.” See the upper part of Fig. 1 for the example of presentation order of Chinese visual SL and English visual SL described below.

## *English visual SL*

Modeled after Kidd et al. (2020), 12 written English syllables (ki, bu, du, mo, di, pa, ta, ka, po, lo, ma, and ri) were used to create four triplets (kibudu, modipa, takapo, and lomari). These syllables were visually presented in a sequence on the screen. Foils were constructed from the same set of syllables (e.g., the foil taburi consisted of syllables taken from the three target triplets takapo, kibudu, and lomari) but crucially differed from the targets in their statistical structure. The four-alternative forced-choice foils were taburi, kimapo, mokari, and lodidu. The English visual SL procedure was the same as the Chinese visual SL procedure.

**Table 1**
Examples of pseudo-characters in Chinese visual statistical learning task.

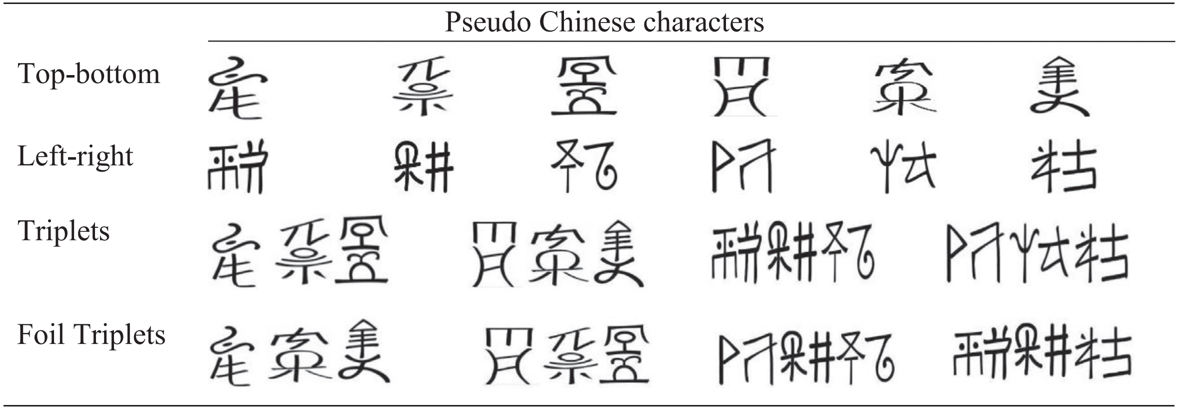

### Non-linguistic auditory SL

Modeled after Qi et al. (2019), 12 pure tones within the same octave (a full chromatic scale starting from middle C) were divided into four base triplets (FGD, G#C#B, CF#D, and EAA#). For the 2AFC task, four additional foils were created: F#BF, AA#G#, C#D#E, and GDC.

Children saw and heard, "Hi! We're going to listen to some alien folk music today. We need you to help us keep track of a special sound as you hear different sounds." In the familiarization phase, we repeated each of the four triplets 48 times for a total of 192 triplets (twice as many as the visual conditions, following the standard paradigm). The duration of each tone was 460 ms with a 20-ms interstimulus interval. Children were instructed to press the spacebar as quickly as possible whenever they heard the target sound in the familiarization phase. The target sound was always the third one of the four base triplets (see the lower part of Fig. 1 for the example of sound presentation order for non-linguistic auditory SL, English auditory SL, and Chinese auditory SL tasks described below). At the beginning of the test phase, children heard and saw, "Now you are going to hear two groups of sound, three as a group. Please select the group of sounds that always followed each other you heard before." Participants were asked to identify which of the two triplets seemed more like what they heard in the familiarization phase.

### English auditory SL

The artificial language used in this experiment was adapted from Schneider et al. (2020) and consisted of 12 English spoken syllables: pa, bi, ku, go, la, tu, da, ro, pi, ti, bu, and do. These were used to construct four trisyllabic-based words: pabiku, golatu, daropi, and tibudo. For 2AFC, an additional four non-word foils were created using the same syllables as the input language. The four foil words were gobutu, parodo, tilapi, and dabiku. The English auditory SL procedure was the same as the non-linguistic auditory SL procedure.

### Chinese auditory SL

Chinese auditory syllables were made comparable to English with four tones evenly distributed across syllables: pā, bí, kù, gōu, lǎ, tù, dě, ròu, pī, tī, bú, and dòu. The four trisyllabic words were pā bíkù, gōulǎtù, děròupī, and tībúdòu. For 2AFC, the four non-word foils were gōubútù, pāróudòu, tīlǎpì, and děbíkù. The Chinese auditory SL procedure was the same as the English auditory SL procedure.

### English receptive vocabulary

To evaluate participants' current English language proficiency, a shortened version of the Peabody Picture Vocabulary Test–Fourth Edition (PPVT; Dunn & Dunn, 2007) was administered to all the participants in the classroom as a group. To maintain the same progression of item difficulty as the original test, 30 items from the original test were selected (Items 1–6, 13–18, 37, 39–43, 49–53, 61–64, 73, and 75–76). The experimenter played a recording of each item once, and children circled the picture in their response booklets that best described the word presented.

### Procedure

Two sessions were involved, each lasting around 35 min. Children completed three SL tasks in each session separated by a 1-week interval. In the first session, children completed non-linguistic visual, Chinese visual, and Chinese auditory SL tasks and an English proficiency test. In the second session, children completed non-linguistic auditory, English visual, and English auditory SL tasks. For each age group, the order of completing SL tasks was counterbalanced within each session. There were six different possible orders in each session, so children were roughly divided into six groups and received one of the orders. In addition, half the children received Session 1 first and the other half received Session 2 first for each age group.

## Results

Arciuli and Simpson (2011) suggested that participants who failed to identify half the targets in the familiarization phase should be excluded from data analysis. All participants in the current study were retained for data analysis because their scores ranged from 67 % to 100 %, and their performance in the familiarization phase did not differ across SL tasks ($p$ >.05). Table 2 lists the accuracy rates for all SL tasks across ages. All participants' SL accuracy rates were within 2.5 standard deviations. Children across all ages performed significantly better than chance level (50 %) across all SL tasks, including non-linguistic visual SL, non-linguistic auditory SL, Chinese visual SL, Chinese auditory SL, English visual SL, and English auditory SL tasks (all $ps$ <.05). Children also appeared to improve their SL with age in all the tasks (see Fig. 2). The mean accuracy of SL tasks in Session 1 was .646 ($SD$ =.14) and in

**Table 2**
Statistical learning accuracy rates across tasks by grade.

| | 9-year-olds | | | 11-year-olds | | | 13-year-olds | | |
|---|---|---|---|---|---|---|---|---|---|
| | Mean | *SD* | *t* | Mean | *SD* | *t* | Mean | *SD* | *t* |
| Non-linguistic visual | .62 | .12 | 6.35*** | .67 | .16 | 6.74*** | .81 | .14 | 13.3*** |
| Non-linguistic auditory | .56 | .07 | 5.28*** | .60 | .12 | 5.16*** | .69 | .13 | 9.07*** |
| English visual | .61 | .10 | 5.82*** | .67 | .13 | 8.24*** | .71 | .17 | 7.74*** |
| English auditory | .54 | .07 | 3.24** | .59 | .11 | 5.06*** | .67 | .14 | 7.32*** |
| Chinese visual | .59 | .11 | 4.67*** | .65 | .13 | 6.97*** | .71 | .14 | 9.03*** |
| Chinese auditory | .62 | .11 | 7.02*** | .64 | .18 | 5.76*** | .73 | .16 | 8.71*** |

*Note.* A total of 40 children were tested in each grade for each task.
** $p$ <.01.
*** $p$ <.001.

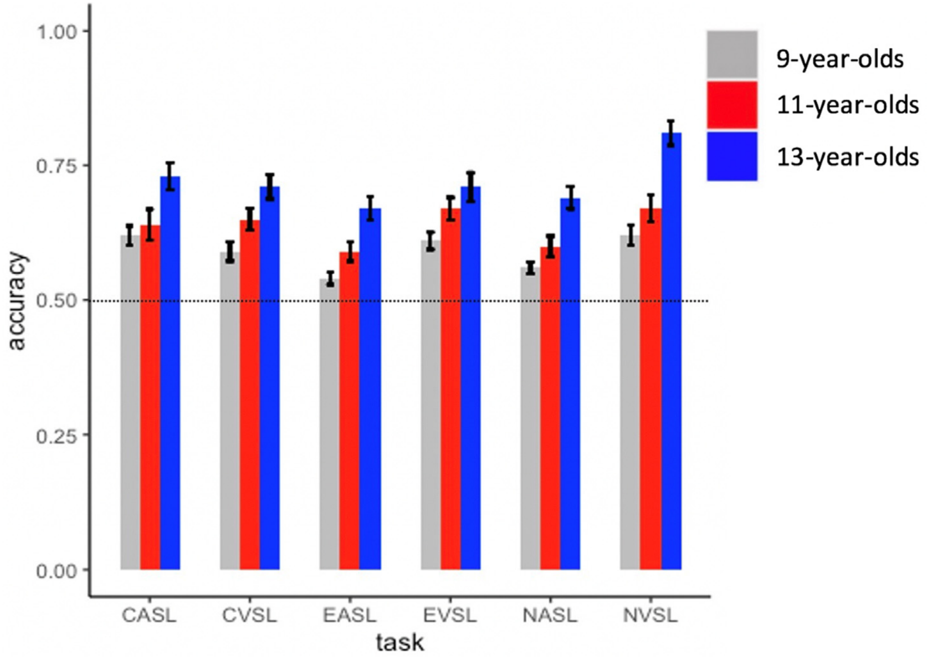


**Fig. 2.** Mean statistical learning (SL) accuracy rates across all the tasks by age. Accuracy rates in the two-alternative forced-choice task are shown. The dotted line represents chance level. CASL, Chinese auditory SL; CVSL, Chinese visual SL; EASL, English auditory SL; EVSL, English visual SL; NASL, non-linguistic auditory SL; NVSL, non-linguistic visual SL.

Session 2 was .649 (*SD* =.15). The mean accuracy did not differ across sessions ($p$ =.76). Children's performance was highly stable across the two sessions ($r$ =.62, $t$ = 8.66, $p$ <.001). The internal consistency reliabilities (i.e., Cronbach's alpha coefficients) of SL tasks were.74 for non-linguistic visual,.76 for non-linguistic auditory,.90 for English visual,.89 for English auditory,.81 for Chinese visual, and.83 for Chinese auditory.

We used a mixed effects logistic regression model (Barr et al., 2013) to examine the effects of age, modality, and linguistic type in the statistical programming language R (R Core Team, 2017). Our dependent binominal variable was success in a single test trial. We started with a full model with a maximum random effects structure justified by the experimental design and removed random factors until the model converged (Barr et al., 2013). The final model included fixed effects for age (centered), modality (visual vs auditory), linguistic type (non-linguistic vs Chinese vs English), the interactions among the three independent variables, and a random intercept for participants (see Table 3). Results showed that the two-way interaction of age and modality was significant ($b$ = 0.14, *SE* = 0.06, $z$ = 2.46, $p$ =.014). Post hoc comparisons revealed that visual SL was associated with a significant increase from 9 to 11 years of age ($b$ = 0.25, *SE* = 0.10, $z$ = 2.55, $p$ =.01) and a significant increase from 11 to 13 years ($b$ = 0.47, *SE* = 0.10, $z$ = 4.67, $p$ = 3.02e-06). However, the increase in the auditory SL accuracy from 9 to 11 years was not significant ($b$ = 0.14, *SE* = 0.10, $z$ = 1.46, $p$ =.145), whereas there was a significant increase from 11 to 13 years ($b$ = 0.44, *SE* = 0.10, $z$ = 4.45, $p$ = 8.66e-06).

The two-way interaction of modality and linguistic type was also significant ($b$ = − 0.15, *SE* = 0.05, $z$ = − 2.93, $p$ =.003). Post hoc comparisons revealed that whereas children performed significantly better in non-linguistic visual than in non-linguistic auditory ($b$ = 1.25, *SE* = 0.15, $z$ = 8.28, $p$ = 2.22e-16) and performed significantly better in English visual than in English auditory ($b$ = 0.88, *SE* = 0.15, $z$ = 6.06, $p$ = 1.38e-9), there was no difference in children's performance across Chinese visual and auditory ($b$ = − 0.16, *SE* = 0.15, $z$ = − 1.08, $p$ =.278). Both the two-way interaction between age and linguistic type and the three-way interaction among age, modality, and linguistic type were not significant ($b$ = − 0.02, *SE* = 0.03, $z$ = − 0.71, $p$ =.475, and $b$ = − 0.07, *SE* = 0.04, $z$ = − 1.69, $p$ =.092, respectively).

The main effects of age, modality, and linguistic type all were significant ($b$ = 0.31, *SE* = 0.06, $z$ = 5.38, $p$ = 7.39e-8, $b$ = 0.31, *SE* = 0.07, $z$ = 4.51, $p$ = 6.42e-6, and $b$ = 0.11, *SE* = 0.04, $z$ = 3.06, $p$ =.002, respectively). Overall, 13-year-olds performed significantly better than 11-year-olds ($b$ = 2.71, *SE* = 0.55, $z$ = 4.89, $p$ = 1.02e-06), 11-year-olds performed significantly better than 9-year-olds ($b$ = − 1.17, *SE* = 0.55, $z$ = − 2.13, $p$ =.033), and 13-year-olds performed significantly better than 9-year-olds ($b$ = 3.88, *SE* = 0.55, $z$ = 7.01, $p$ = 2.33e-12). Participants overall performed significantly better in visual SL than in auditory SL, performed better in non-linguistic SL than in English SL ($b$ = 0.97, *SE* = 0.21, $z$ = 4.62, $p$ = 3.84e-6), and performed better in Chinese SL than in English SL ($b$ = 0.68, *SE* = 0.21, $z$ = 3.29, $p$ =.001). Nevertheless, the difference in participants' performance between non-linguistic SL and Chinese SL was not significant ($b$ = 0.29, *SE* = 0.21, $z$ = 1.36, $p$ =.174).

To examine the effect of Chinese orthographic structure on Chinese visual SL accuracy, we ran another mixed effects logistic regression model, including Chinese orthographic structure

**Table 3**
Regression model for statistical learning across age groups, modalities, and linguistic types.

| | Estimate | *SE* | *z* Value | *p* Value |
|---|---|---|---|---|
| Intercept | .14 | .07 | 1.91 | .05677 |
| Age group | .31 | .06 | 5.38 | 7.43e-8*** |
| Modality | .31 | .07 | 4.51 | 6.42e-6*** |
| Language | .11 | .04 | 3.06 | .00221** |
| Age Group × Modality | .14 | .06 | 2.46 | .01382* |
| Age Group × Linguistic Type | −.02 | .03 | −0.71 | .47527 |
| Modality × Linguistic Type | −.15 | .05 | −2.93 | .00337** |
| Age × Modality × Linguistic Type | −.07 | .04 | −1.69 | .09169 |

* $p$ <.05.
** $p$ <.01.
*** $p$ <.001.

(top–bottom vs left–right), age (centered), and the interaction between them as fixed factors. The model had a random intercept for participants, and Chinese visual SL accuracy in each trial served as a dependent binominal variable. Results showed that there was a significant interaction between Chinese orthographic structure and age ($b = 0.11$, $SE = 0.04$, $z = 2.54$, $p = .011$). Post hoc comparisons revealed that whereas 9- and 11-year-olds performed significantly better in Chinese top–bottom structure than in left–right structure ($b = 0.40$, $SE = 0.12$, $z = 3.42$, $p = .001$, and $b = 0.50$, $SE = 0.12$, $z = 4.13$, $p = 3.66e-5$, respectively), 13-year-olds did not perform differently between the two Chinese orthographic structures ($b = -0.06$, $SE = 0.13$, $z = -0.45$, $p = .657$). The main effects of Chinese orthographic structure and age were also significant ($b = -0.28$, $SE = 0.07$, $z = -4.03$, $p = 5.61e-5$, and $b = 0.09$, $SE = 0.04$, $z = 2.26$, $p = .024$, respectively). Children overall performed better in the top–bottom structure than in the left–right structure. The 13-year-olds performed significantly better than the 11-year-olds ($b = -0.63$, $SE = 0.28$, $z = -2.22$, $p = .026$), 11-year-olds performed significantly better than 9-year-olds ($b = -0.56$, $SE = 0.28$, $z = -2.03$, $p = .043$), and 13-year-olds performed significantly better than 9-year-olds ($b = -1.19$, $SE = 0.28$, $z = -4.24$, $p = 2.23e-5$).

Finally, English vocabulary and English visual SL were significantly correlated with each other ($r = .42$, $p < .001$), and so were English vocabulary and English auditory SL ($r = .50$, $p < .001$). Furthermore, English vocabulary and Chinese visual SL were significantly correlated with each other ($r = .42$, $p < .001$), and so were English vocabulary and Chinese auditory SL ($r = .41$, $p < .001$). English vocabulary and non-linguistic visual SL were significantly correlated with each other ($r = .49$, $p < .001$), and so were English vocabulary and non-linguistic auditory SL ($r = .51$, $p < .001$).

## Discussion

The goal of the current study was to investigate the effects of modality (visual vs auditory) and linguistic type (non-linguistic vs Chinese vs English) in the developmental trajectory of SL across children aged 9, 11, and 13 years. There were several important findings. First, we showed three main effects of age, modality, and linguistic type. Overall, children developed their SL abilities across the three age groups, and they performed better in visual modality than in auditory modality. Moreover, children performed better in non-linguistic and Chinese SL compared with English SL. Most important, we showed a significant interaction between age and modality and that the developmental trajectory was different across visual and auditory modalities. Whereas visual SL constantly improved from 9 to 11 to 13 years of age, auditory SL improved only from 11 to 13 years but not from 9 to 11 years. The same pattern of the age group and modality interaction occurred across non-linguistic, Chinese, and English SL. A significant interaction between modality and linguistic type was shown, and that better learning was achieved in visual as compared with auditory SL in both non-linguistic and English stimuli. However, children performed similarly across the two modalities in Chinese. The same pattern of modality and linguistic type interaction occurred across 9-, 11-, and 13-year-olds. In addition, whereas children aged 9 and 11 years demonstrated better learning in Chinese top–bottom structure, 13-year-olds did not show this learning bias. Finally, all the correlations between English vocabulary and SL measures were significant.

### *The interaction between age and modality*

Our findings were consistent with our hypotheses and those in previous studies in terms of the significant improvement of non-linguistic visual SL during childhood (e.g., Arciuli & Simpson, 2011; Raviv and Arnon, 2018; Shufaniya & Arnon, 2018). For example, Raviv and Arnon (2018) tested children aged 5 to 12 years on matching visual and auditory SL tasks, and they also showed a development trajectory of visual SL in the non-linguistic domain. This finding suggests that the developmental nature of non-linguistic visual SL is robust during childhood. More important, our study is one of the first to demonstrate that the developmental nature of visual SL is not specific to non-linguistic input, but it also applies to linguistic stimuli, including both Chinese L1 and English L2. This finding is contradictory to the speculation by Shufaniya and Arnon (2018) that linguistic visual SL, like linguistic auditory SL, might not grow with age due to its linguistic nature. Our finding suggests that children across early,

middle, and late childhood can extract the statistical patterns from visual input regardless of the domain in which it is presented (images, English syllables, or Chinese characters), and this skill increases with age.

The auditory modality, however, was different from our hypotheses and previous findings, which mentioned that only non-linguistic auditory SL increases as a function of age and linguistic auditory SL appears to be age invariant (Shufaniya & Arnon, 2018). Our study demonstrated that, regardless of auditory stimuli type (non-linguistic, Chinese L1, or English L2), auditory SL increased from 11 to 13 years of age but not from 9 to 11 years. Shufaniya and Arnon (2018) argued that the auditory knowledge of syllable co-occurrence has already matured during early childhood (see also Storkel, 2001), so the ability of extracting linguistic auditory SL information will not change after early childhood. Why, then, did our data show that there is still a developmental improvement from 11 to 13 years of age? One possible explanation for this inconsistent finding may lie in the difference of presentation speed between auditory stimuli (460 ms per stimulus) and visual stimuli (800 ms per stimulus) in the current study. Emberson et al. (2011) revealed that auditory and visual SL have opposite timing constraints, where visual SL performs better in slow presentation and auditory SL performs better in fast presentation. Both slow visual and fast auditory presentation rates may demand less attention and working memory, whereas fast visual and slow auditory presentation rates may demand greater attention and working memory. Multiple works have shown that SL should be thought of as a componential theoretical construct rather than a unified one, and attention and working memory can be underlying components of SL (e.g., Arciuli, 2017; Arciuli & Conway, 2018). Note that the stimulus onset asynchrony (i.e., each stimulus + interstimulus rate) in our study was very slow—1000 ms compared with other studies (e.g., 600 ms in Arciuli & Simpson, 2011; 500 ms in Bertels et al., 2015; 600 ms in Raviv & Arnon, 2018)—so our visual SL may require relatively less attention and working memory; however, our auditory presentation rate was midway (480 ms) between how quickly they can be presented (e.g., 222 ms in Saffran et al., 1996, 1997) and how slowly they could be presented (e.g., 750 ms in Emberson et al., 2011). Therefore, we speculate that, compared with the slow presentation of our visual stimuli, the relatively faster auditory stimuli in the auditory SL task may demand more attention and working memory to help encode and process the information. Furthermore, as suggested by Broadbent et al. (2017, 2018), the use of visual information may help learners to focus their attention on the visual features of a learning task, leading to better incidental learning of category information. In our study, the presence of visual symbols may also serve as an aid to focus attention and reduce working memory load in the visual SL tasks. Taken together, these may help to explain why we did not observe a significant change from 9 to 11 years of age, but a significant increase in children's auditory SL was evident from 11 to 13 years. Future research would benefit from having a working memory task to directly measure working memory in children across these ages alongside SL tasks and to see whether it is associated with children's SL across modalities.

### *The interaction between modality and language*

Our results showed that children performed better in visual SL compared with auditory SL in non-linguistic and English SL. The visual advantage shown in our study is similar to the findings from Siegelman and Frost (2015), Raviv and Arnon (2018), and Shufaniya and Arnon (2018). Raviv and Arnon (2018) argued that the visual advantage in their study could be due to the fact that the alien visual stimuli is more attractive than auditory sounds and is easier to encode for children. Nevertheless, our study showed that the visual advantage was not limited to non-linguistic alien stimuli but also to English syllable stimuli. One possible explanation is that native Chinese-speaking children, as L2 learners of English, rely more on visual information compared with auditory information in learning and processing English words (e.g., Wang & Geva, 2003), and Chinese language teachers of our participants put more emphasis on recognizing visual patterns of English syllables than on listening and speaking skills, based on our survey. The consequence of this teaching style is that children performed much better in reading and writing than in listening and speaking in English (Zhou, 2005).

For Chinese SL, on the other hand, there was no significant difference between Chinese visual and auditory SL, which is contrary to our hypothesis. The average accuracy rates of Chinese visual SL and English visual SL were similar, but children performed much better in Chinese auditory SL than in

English auditory SL. One explanation is that our children had a relatively weaker auditory SL in L2 compared with that in L1, which is not surprising given that English is their L2. Another potential explanation is that the presence of tonal information in the auditory Chinese stimuli improves Chinese auditory SL, thereby minimizing the advantage of visual SL given that the syllable structures and phoneme types are very similar across the Chinese and English linguistic stimuli, and the only difference is the presence of tones in the Chinese auditory stimuli. Our hypothesis is that not only do children rely on syllable segmental information to detect the correct sequence of the triplets in Chinese, but they also use the additional tonal information superimposed onto the segmental sounds for the statistical pattern identification. Future research is needed to test this hypothesis directly.

### *The effect of character structure and the role of English proficiency*

The finding that children aged 9 and 11 years learn Chinese top–bottom structure better than left–right structure is in accordance with that in Tong and McBride (2014), where children's invented character production accuracy rate was higher for the top–bottom structure than for the left–right structure, and children made more positional errors on the left–right structure than on the top–bottom structure. The first explanation offered by Tong and McBride (2014) on this top–bottom structure preference was the greater prevalence of the left–right structure (>70 %) compared with the top–bottom structure in the Chinese writing system, and children tend to pay more attention to the less common structure. The second explanation was that the position of stroke patterns embedded in top–bottom structured characters is highly predictable and easier to learn. There are a relatively small portion of top–bottom structured characters, and the stroke patterns making up the top–bottom characters are less extensive. But why does this learning bias disappear by 13 years of age in our study? We speculate that after 11 years children's Chinese character reading is much enhanced in both top–bottom and left–right structured characters, and the preference for a particular type of structure fades gradually.

In addition, we showed a significant positive correlation between all six types of SL and English L2 vocabulary. This result supports and extends previous findings on the important role of different forms of SL in vocabulary acquisition. SL has been shown to be one important early contributor to vocabulary development (e.g., Evans et al., 2009; Hedenius et al., 2011; Kemény & Lukács, 2021; Reuter et al., 2018; Saffran et al., 2009). For example, Evans et al. (2009) revealed a link between auditory SL and language proficiency including vocabulary in children aged 6 to 14 years. Kemény and Lukács (2021) demonstrated that SL skills had an independent contribution to vocabulary size over and above age, receptive grammatical ability, and short-term memory during childhood. Our finding further suggests that SL in L2 across modalities and domains is strongly associated with children's L2 vocabulary knowledge. This association is consistent with the hypothesis that SL plays a critical role in word segmentation. Word segmentation is crucial in lexical development (Erickson & Thiessen, 2015). After all, lexical acquisition not only in L1 but also in L2 relies on distributional information and is supported by SL.

## Limitations

There are several limitations in the current study. First, we only investigated children aged 9, 11, and 13 years. It would be valuable to track SL performance across modalities and domains across a greater range of age such as from infancy to late childhood. To achieve this goal, researchers need to be able to design an SL paradigm suitable for both infants and children. Second, as a cross-sectional study, our research was not able to detect individual changes in SL ability. Longitudinal studies are extremely helpful in this area that sets out to provide a more comprehensive understanding of individual differences in the developmental trajectory of SL. In addition, the different patterns of SL between Chinese and English could be a combined effect of the writing system (alphabetic vs non-alphabetic) and language status (L1 vs L2). Our current sample of participants would not allow us to tease these two effects apart cleanly. Future research needs to recruit monolingual Chinese-speaking and monolingual English-speaking children for a more direct comparison in terms of their

linguistic SL to address the language effect. Moreover, we did not include a measure of reading fluency as a predictor to allow us to address the role of individual differences in reading skills in developing SL. Future research may include this measure to address this question. Finally, the three syllables in each English triplet are equally stressed without a specific stress assignment in the English auditory SL task. This may result in unnatural English spoken multisyllabic words. Future research needs to consider a specific stress assignment in each triplet in the auditory English SL task to make it comparable with the Chinese triplets in the auditory Chinese SL task in terms of the presence of the supra-segmental information.

## Conclusions

Our study makes a unique contribution to the growing body of literature on the development of SL across visual and auditory modalities in non-linguistic and linguistic domains. Our findings suggest that the developmental trajectory of SL differs across visual and auditory modalities. Whereas visual SL constantly improves from 9 to 11 to 13 years of age, auditory SL only improves significantly from 11 to 13 years, probably due to the faster temporal processing speed in auditory stimuli because it requires a higher demand on attention and working memory. Furthermore, visual modality advantage was shown in non-linguistic and English SL but not in Chinese SL, possibly due to the contribution from tonal information. Together, our findings point to the joint function of age, modality, and linguistic type in SL development.

## Data availability

Data will be made available on request.

## Acknowledgments

This work was supported by the Graduate Research Funding awarded to the first author at the Department of Human Development and Quantitative Methodology, University of Maryland, College Park. We thank Yang Liu at the University of Maryland, College Park, for his kind guidance on the statistical analyses. We are grateful to all the teachers for their help with recruiting the participants. We are also grateful to the children who participated in this study.

## References

Arciuli, J. (2017). The multi-component nature of statistical learning Article 20160058. *Philosophical Transactions of the Royal Society B: Biological Sciences, 372*(1711). https://doi.org/10.1098/rstb.2016.0058.

Arciuli, J., & Conway, C. M. (2018). The promise—and challenge—of statistical learning for elucidating atypical language development. *Current Directions in Psychological Science, 27*(6), 492–500. https://doi.org/10.1177/0963721418779977.

Arciuli, J., & Simpson, I. C. (2011). Statistical learning in typically developing children: The role of age and speed of stimulus presentation. *Developmental Science, 14*(3), 464–473. https://doi.org/10.1111/j.1467-7687.2009.00937.x.

Barr, D. J., Levy, R., Scheepers, C., & Tily, H. J. (2013). Random effects structure for confirmatory hypothesis testing: Keep it maximal. *Journal of Memory and Language, 68*(3), 255–278. https://doi.org/10.1016/j.jml.2012.11.001.

Bertels, J., Boursain, E., Destrebecqz, A., & Gaillard, V. (2015). Visual statistical learning in children and young adults: How implicit? Article 1541 *Frontiers in Psychology, 5*. https://doi.org/10.3389/fpsyg.2014.01541.

Broadbent, H. J., Osborne, T., Rea, M., Peng, A., Mareschal, D., & Kirkham, N. Z. (2018). Incidental category learning and cognitive load in a multisensory environment across childhood. *Developmental Psychology, 54*(6), 1020–1028. https://doi.org/10.1037/dev0000472.

Broadbent, H. J., White, H., Mareschal, D., & Kirkham, N. Z. (2017). Incidental learning in a multisensory environment across childhood. *Developmental Science, 21*(2). https://doi.org/10.1111/desc.12554.

Ceccagno, A., & Basciano, B. (2007, January). Classification of Chinese compounds. In G. Booij, A. Ralli, & S. Scalise (Eds.), *Proceedings of the Sixth Mediterranean Morphology Meeting* (Vol. 6, pp. 71–83).

Chao, Y. R. (1968). *Grammar of spoken Chinese*. University of California Press.

de Leeuw, J. R. (2015). jsPsych: A JavaScript library for creating behavioral experiments in a web browser. *Behavior Research Methods, 47*(1), 1–12. https://doi.org/10.3758/s13428-014-0458-y.

Dunn, L. M., & Dunn, D. M. (2007). *PPVT-4*. Peabody Picture Vocabulary Test. Pearson Assessments.

Emberson, L. L., Conway, C. M., & Christiansen, M. H. (2011). Timing is everything: Changes in presentation rate have opposite effects on auditory and visual implicit statistical learning. *Quarterly Journal of Experimental Psychology, 64*(5), 1021–1040. https://doi.org/10.1080/17470218.2010.538972.

Emberson, L. L., Misyak, J. B., Schwade, J. A., Christiansen, M. H., & Goldstein, M. H. (2020). Comparing statistical learning across perceptual modalities in infancy: An investigation of underlying learning mechanism(s) Article e12847. *Developmental Science, 22*(6). https://doi.org/10.1111/desc.12847.

Enochson, K. (2015). *Adaptation as statistical learning: An individual differences study*. George Mason University. Doctoral dissertation,.

Erickson, L. C., & Thiessen, E. D. (2015). Statistical learning of language: Theory, validity, and predictions of a statistical learning account of language acquisition. *Developmental Review, 37*, 66–108. https://doi.org/10.1016/j.dr.2015.05.002.

Evans, J. L., Saffran, J. R., & Robe-Torres, K. (2009). Statistical learning in children with specific language impairment. *Journal of Speech, Language, and Hearing Research, 52*, 321–335 https://doi:10.1044/1092-4388(2009/07-0189.

He, X., & Tong, X. (2017). Statistical learning as a key to cracking Chinese orthographic codes. *Scientific Studies of Reading, 21*(1), 60–75. https://doi.org/10.1080/10888438.2016.1243541.

Hedenius, M., Persson, J., Tremblay, A., Adi-Japha, E., Veríssimo, J., Dye, C. D., Alm, P., Jennische, M., Bruce Tomblin, J., & Ullman, M. T. (2011). Grammar predicts procedural learning and consolidation deficits in children with specific language impairment. *Research in Developmental Disabilities, 32*(6), 2362–2375 https://doi-org.ezproxy.its.uu.se/10.1016/j.ridd.2011.07.026.

Isbilen, Erin S. et al (2020). Statistically Induced Chunking Recall: A Memory-Based Approach to Statistical Learning. *Cognitive Science, 44*(7). https://doi.org/10.1111/cogs.12848.

Kemény, F., & Lukács, Á. (2021). The role of statistical learning and verbal short-term memory in impaired and typical lexical development. *Frontiers in Communication, 6*. https://doi.org/10.3389/fcomm.2021.700452 700452.

Kidd, E., Arciuli, J., Christiansen, M. H., Isbilen, E. S., Revius, K., & Smithson, M. (2020). Measuring children's auditory statistical learning via serial recall. *Journal of Experimental Child Psychology, 200*. https://doi.org/10.1016/j.jecp.2020.104964 104964.

Kirkham, N. Z., Slemmer, J. A., & Johnson, S. P. (2002). Visual statistical learning in infancy: Evidence for a domain general learning mechanism. *Cognition, 83*(2), B35–B42. https://doi.org/10.1016/s0010-0277(02)00004-5.

Lee, S. M. K., & Tong, X. (2020). Spelling in developmental dyslexia in Chinese: Evidence of deficits in statistical learning and over-reliance on phonology. *Cognitive Neuropsychology, 37*(7–8), 494–510. https://doi.org/10.1080/02643294.2020.1765754.

Qi, Z., Sanchez Araujo, Y., Georgan, W. C., Gabrieli, J. D., & Arciuli, J. (2019). Hearing matters more than seeing: A cross-modality study of statistical learning and reading ability. *Scientific Studies of Reading, 23*(1), 101–115. https://doi.org/10.1080/10888438.2018.1485680.

R Core Team (2017). *R: A language and environment for statistical computing.* R Foundation for Statistical Computing. https://www.R-project.org.

Raviv, L., & Arnon, I. (2018). The developmental trajectory of children's auditory and visual statistical learning abilities: Modality-based differences in the effect of age Article e12593. *Developmental Science, 21*(4). https://doi.org/10.1111/desc.12593.

Reuter, T., Emberson, L., Romberg, A., & Lew-Williams, C. (2018). Individual differences in nonverbal prediction and vocabulary size in infancy. *Cognition, 176*, 215–219. https://doi.org/10.1016/j.cognition.2018.03.006.

Saffran, J. R., Aslin, R. N., & Newport, E. L. (1996). Statistical learning by 8-month-old infants. *Science, 274*(5294), 1926–1928. https://doi.org/10.1126/science.274.5294.1926.

Saffran, J. R., Johnson, E. K., Aslin, R. N., & Newport, E. L. (1999). Statistical learning of tone sequences by human infants and adults. *Cognition, 70*(1), 27–52. https://doi.org/10.1016/s0010-0277(98)00075-4.

Saffran, J. R., & Kirkham, N. Z. (2018). Infant statistical learning. *Annual Review of Psychology, 69*, 181–203. https://doi.org/10.1146/annurev-psych-122216-011805.

Saffran, J. R., Newport, E. L., Aslin, R. N., Tunick, R. A., & Barrueco, S. (1997). Incidental language learning: Listening (and learning) out of the corner of your ear. *Psychological Science, 8*(2), 101–105. https://doi.org/10.1111/j.1467-9280.1997.tb00690.x.

Saffran, J. R., Pelucchi, B., & Hay, J. F. (2009). Statistical learning in a natural language by 8-month-old infants. *Child Development, 80*(3), 674–685. https://doi.org/10.1111/j.1467-8624.2009.01290.x.

Schlichting, M. L., Guarino, K. F., Schapiro, A. C., Turk-Browne, N. B., & Preston, A. R. (2017). Hippocampal structure predicts statistical learning and associative inference abilities during development. *Journal of Cognitive Neuroscience, 29*(1), 37–51. https://doi.org/10.1162/jocn_a_01028.

Schneider, J. M., Hu, A., Legault, J., & Qi, Z. (2020). Measuring statistical learning across modalities and domains in school-aged children via an online platform and neuroimaging techniques Article e61474. *JoVE (Journal of Visualized Experiments), 160*. https://doi.org/10.3791/61474.

Shu, H., & Anderson, R. C. (1997). Role of radical awareness in the character and word acquisition of Chinese children. *Reading Research Quarterly, 32*(1), 78–89. https://doi.org/10.1598/RRQ.32.1.5.

Shu, H., Chen, X., Anderson, R. C., Wu, N., & Xuan, Y. (2003). Properties of school Chinese: Implications for learning to read. *Child Development, 74*(1), 27–47. https://doi.org/10.1111/1467-8624.00519.

Shufaniya, A., & Arnon, I. (2018). Statistical learning is not age-invariant during childhood: Performance improves with age across modality. *Cognitive Science, 42*(8), 3100–3115. https://doi.org/10.1111/cogs.12692.

Siegelman, N., & Frost, R. (2015). Statistical learning as an individual ability: Theoretical perspectives and empirical evidence. *Journal of Memory and Language, 81*, 105–120. https://doi.org/10.1016/j.jml.2015.02.001.

Storkel, H. L. (2001). Learning new words: Phonotactic probability in language development. *Journal of Speech, Language, and Hearing Research, 44*(6), 1321–1337. https://doi.org/10.1044/1092-4388(2001/103).

Teinonen, T., Fellman, V., Näätänen, R., Alku, P., & Huotilainen, M. (2009). Statistical language learning in neonates revealed by event-related brain potentials Article 21. *BMC Neuroscience, 10*(1). https://doi.org/10.1186/1471-2202-10-21.

Tong, X., & McBride, C. (2014). Chinese children's statistical learning of orthographic regularities: Positional constraints and character structure. *Scientific Studies of Reading, 18*(4), 291–308. https://doi.org/10.1080/10888438.2014.884098.

Wang, M., & Geva, E. (2003). Spelling performance of Chinese children using English as a second language: Lexical and visual–orthographic processes. *Applied Psycholinguistics, 24*(1), 1–25. https://doi.org/10.1017/S0142716403000018.

Wang, T., & Saffran, J. R. (2014). Statistical learning of a tonal language: The influence of bilingualism and previous linguistic experience Article 953. *Frontiers in Psychology, 5*. https://doi.org/10.3389/fpsyg.2014.00953.

Yang, X., & Meng, X. (2020). Visual processing matters in Chinese reading acquisition and early mathematics Article 462. *Frontiers in Psychology, 11*. https://doi.org/10.3389/fpsyg.2020.00462.

Yin, L., & McBride, C. (2015). Chinese kindergartners learn to read characters analytically. *Psychological Science, 26*(4), 424–432. https://doi.org/10.1177/0956797614567203.

Zhou, N. (2005). *How English as a second language affects Chinese students giving presentations during class in US*. Marietta College. Doctoral dissertation.

Zhou, Y. (2014). Writing evolution initiated by Naxi-Sino writing contact. *Studies in Literature and Language, 8*(3), 156–159.
